# A new prognostic risk model based on autophagy-related genes in kidney renal clear cell carcinoma

**DOI:** 10.1080/21655979.2021.1976050

**Published:** 2021-10-12

**Authors:** Guangzhen Wu, Yingkun Xu, Huayu Zhang, Zihao Ruan, Peizhi Zhang, Zicheng Wang, Han Gao, Xiangyu Che, Qinghua Xia, Feng Chen

**Affiliations:** a Department of Urology, The First Affiliated Hospital of Dalian Medical University, Dalian, China; b Department of Urology, Shandong Provincial Hospital, Cheeloo College of Medicine, Shandong University, Jinan, China; c Department of Plastic and Reconstructive Surgery, Shandong Qianfoshan Hospital, Cheeloo College of Medicine, Shandong University, Jinan, China; d Department of Nursing, Zhengzhou University, Zhengzhou, China; e Department of Urology, Shandong Provincial Hospital Affiliated to Shandong First Medical University, Jinan, China

**Keywords:** Autophagy, kidney renal clear cell carcinoma, tcga, prognostic risk model, nomogram

## Abstract

This study aimed to explore the potential role of autophagy-related genes in kidney renal clear cell carcinoma (KIRC) and develop a new prognostic-related risk model. In our research, we used multiple bioinformatics methods to perform a pan-cancer analysis of the CNV, SNV, mRNA expression, and overall survival of autophagy-related genes, and displayed the results in the form of heat maps. We then performed cluster analysis and LASSO regression analysis on these autophagy-related genes in KIRC. In the cluster analysis, we successfully divided patients with KIRC into five clusters and found that there was a clear correlation between the classification and two clinicopathological features: tumor, and stage. In LASSO regression analysis, we used 13 genes to create a new prognostic-related risk model in KIRC. The model showed that the survival rate of patients with KIRC in the high-risk group was significantly lower than that in the low-risk group, and that there was a correlation between this grouping and the patients’ metastasis, tumor, stage, grade, and fustat. The results of the ROC curve suggested that this model has good prediction accuracy. The results of multivariate Cox analysis show that the risk score of this model can be used as an independent risk factor for patients with KIRC. In summary, we believe that this research provides valuable data supporting future clinical treatment and scientific research.

## 1. Introduction

In the past few decades, research related to autophagy in the field of tumor medicine has continued. Autophagy is an evolutionarily conserved catabolic process in the cell that can deliver cytoplasmic macromolecules, aggregated proteins, damaged organelles, or pathogens to the lysosome, where they are digested by hydrolytic enzymes in the lysosome to produce nucleotides, amino acids, fatty acids, sugars, and ATP, ultimately recycling the materials [1–3]. The main processes involved in autophagy are initiation (nucleation), elongation-maturation, fusion, and degradation [4–6]. There is a close relationship between autophagy and many human diseases, such as cancer, neurodegenerative diseases, and autoimmune diseases [6,7]. Autophagy is a potential regulator of cell death; thus, it is a therapeutic target for cancer [8–10]. Tumor cells are often associated with abnormal autophagy activity. In the early stage of tumor development, the loss of autophagy function can lead to malignant transformation of cells and can promote tumorigenesis and growth. In the late stage of tumor development, as the tumor volume increases, the cells are in a state of hypoxia and nutritional deprivation. Activation of autophagy as a survival mechanism maintains the ability of tumor cells to survive in unfavorable environments. It is shown that it affects both the time and space required for tumor growth [11–13]. Therefore, cell autophagy is closely related to the occurrence and development of tumors.

Renal cell carcinoma (RCC) is a malignant tumor of the renal tubular epithelial cell system that originates in kidney tissue. It is often referred to as kidney cancer and is one of the most common tumors of the urinary system. Global cancer statistics from 2018 showed that 403,262 new cases of kidney cancer occur each year, with a reported 175,098 deaths [14,15]. There are three main subtypes of RCC: kidney renal clear cell carcinoma (KIRC), kidney renal papillary cell carcinoma (KIRP), and kidney chromophobe (KICH). KIRC accounts for 75–80% of all RCC [16]. As the early clinical symptoms of kidney cancer are relatively difficult to detect, more than 30% of patients have metastasis after diagnosis. Moreover, radiotherapy, chemotherapy, and endocrine treatment of patients with renal cancer are not ideal. Surgery is the only possible cure, but 20% of patients still experience recurrence and metastasis after surgery [17,18]. Therefore, developing an accurate and reliable risk model has become an important research direction to improve the prognosis of renal cancer.

In view of the progress in precision medicine, higher requirements are imposed on clinical diagnosis and treatment. The establishment of prognostic models in clinical cancer management has become increasingly critical because doctors can use these models to intervene promptly in high-risk patients while avoiding the overtreatment of low-risk patients [19]. To improve the accuracy of the prognostic evaluation guidelines in the current practices of clinical diagnosis and treatment, they are continuously modified, while taking into account the ease of use of clinical use [20–22]. In this study, to explore the potential biological role of autophagy-related genes in KIRC, we conducted CNV, SNV, mRNA expression and overall survival analysis for these genes in KIRC. Most importantly, we used cluster analysis to successfully divide KIRC patients into five clusters. In addition, we use LASSO regression analysis to establish a new prognostic-related risk model in KIRC. This model contained 13 genes: ATG4A, GABARAPL2, ATG10, ATG12, ATG2B, ATG4C, ATG5, ULK1, ATG16L2, ATG2A, ATG13, MAP1LC3C, and GABARAP. In previous studies, some researchers have used autophagy-related genes to establish new prognostic-related risk models in esophageal adenocarcinoma and hepatocellular carcinoma [23,24]. Different from these research works, in this study, we used autophagy-related genes to perform a large number of pan-cancer analyses, and used cluster analysis to successfully divide KIRC patients into five clusters. We believe that the results of our study have provided valuable and reliable data for future scientific research and clinical diagnosis and treatment.

## 2. Materials and methods

### 2.1. Data collection

The Cancer Genome Atlas (TCGA) program was launched in 2006 by the National Cancer and Cancer Institute (NCI) and the National Human Genome Institute (NHGRI). The goal of the program is to map cancer gene maps, understand the molecular mechanisms of cancer, and improve our ability to prevent, diagnose, and treat cancer. In this study, CNV, SNV, and mRNA expression profiles and clinical data of pan-cancer transcriptomes were downloaded and compiled from the TCGA database. The KIRC database contains 539 tumor tissues and 72 normal tissues. Then, we collected 29 autophagy-related genes from an important review related to autophagy [1].

### 2.2. Oncomine database

The Oncomine database is a gene chip-based database and integrated data-mining platform, which is used mainly to collect, standardize, analyze cancer transcriptome data and share results within the biomedical research community (https://www.oncomine.org/) [25]. In this study, we used this database to perform a pan-cancer analysis of the expression of autophagy-related genes.

### 2.3. GEPIA website

The GEPIA website is a tool that provides rapid analysis of differential expression, draws contour maps, analyses patient survival, and detects similar genes based on TCGA and GTEx data (http://gepia2.cancer-pku.cn/#index) [26]. In this study, we used the GEPIA database to explore the expression of the candidate gene ATG9B in various tumors.

### 2.4. Generation of PPI networks

The String database analyses known and predicted protein-protein interactions. Currently, the database contains 9,643,763 proteins from 2031 organisms, and has information on direct (physical) and indirect (functional) interactions (https://string-db.org/) [27]. Cytoscape, an open-source bioinformatics software platform, can visualize molecular interaction networks by constructing protein interaction networks [28]. We used the String website to obtain the protein interaction networks of autophagy-related genes, exported the results in TSV format, and imported the resulting source file into Cytoscape for visual analysis.

### 2.5. GSCALite website

GSCALite is a website that integrates TCGA, GDSC, CTRP, and GTEx data for genome analysis. It can be used for dynamic analysis and visualization of the cancer genome and to determine correlations with drug sensitivity. Cancer researchers can use this website for cancer genome analysis (http://bioinfo.life.hust.edu.cn/web/GSCALite/) [29]. We used this tool to explore the relationship between the methylation levels of all autophagy-related genes and the overall survival of patients with multiple tumors, and then analyzed the relationship between these genes and cancer pathways. Finally, the GDSC data from this website were used to analyze the sensitivity between these genes and anticancer drugs.

### 2.6. Data processing and analysis

We downloaded the latest version of the freely available official R software from CRAN (https://www.r-project.org/). As the environment provided by R software is complex to navigate, we used RStudio, which is a simple and powerful R language operation platform (https://www.rstudio.com/). The data processing and data analysis part of this research was performed using Perl and multiple R language packages. The heat map in this study was drawn by running the Pheatmap package, and then further processed by TBtools (https://github.com/CJ-Chen/TBtools). The difference analysis was performed using the Limma package. Co-expression analysis was performed using the Corrplot package. In addition, for the cluster analysis of this study, we mainly used the Consensus Cluster Plus package. LASSO regression analysis was mainly implemented with the help of the Glmnet and Survival packages. The Survival package was used to draw survival curves, and the Survival ROC package was used to analyze and draw ROC curves. Finally, under this risk model, univariate cox analysis and multivariate cox analysis with clinical characteristics were performed. A P-value of <0.05 was considered statistically significant.

## 3. Results

### 3.1. Pan-cancer overview of variation of autophagy-related genes

**Figure 1.**
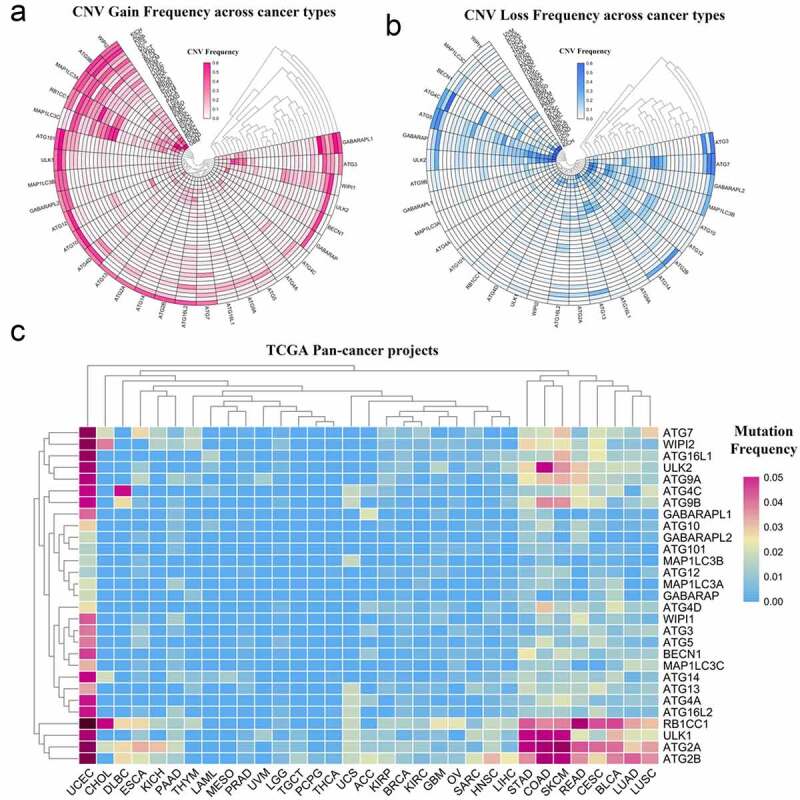
Panoramic view of the mutation of autophagy-related genes in pan-cancer. (a) Copy number variation gain frequency across cancer types. The redder the color, the higher the mutation frequency of the corresponding molecule in corresponding cancer. (b) Copy number variation loss frequency across cancer types. The bluer the color, the higher the loss frequency of the corresponding molecule in corresponding cancer. (c) Single nucleotide variation in pan-cancer. As the frequency of the mutation increases, the color on the small square changes from blue to red

Although many autophagy-related genes have been explored in tumors, the mutations of these genes in a variety of tumors are not well summarized [30,31]. In this study, to perform a pan-cancer investigation of the mutation of autophagy-related genes, we analyzed high-throughput CNV and SNV data collected from the TCGA database and presented it as a heat map. From the response heat map of the CNV gain frequency, it can be seen that autophagy-related genes have higher frequencies of gain mutations in KICH, ACC, KIRP, and OV. WIPI2, ATG9B, MAP1LC3A, and RB1CC1 have higher frequencies of gain mutation frequencies in the pan-cancer analysis (Figure 1a, Table S1). In addition, from the heat map of CNV loss frequency, autophagy-related genes had a higher frequency of loss mutations in UVM, UCS, OV, and KICH. ATG5, GABARAP, ATG7, and ULK2 had a higher frequency of loss mutations in the pan-cancer analysis (Figure 1b, Table S2). In the heat map generated from the SNV data from TCGA, autophagy-related genes had higher frequencies of mutation in UCEC, STAD, COAD, and SKCM. ATG2B, ATG2A, ULK1, and RB1CC1 had a wide range of mutation frequencies in the pan-cancer analysis (Figure 1c, Table S3).

### 3.2. Pan-cancer overview of the mRNA expression of autophagy-related genes

**Figure 2.**
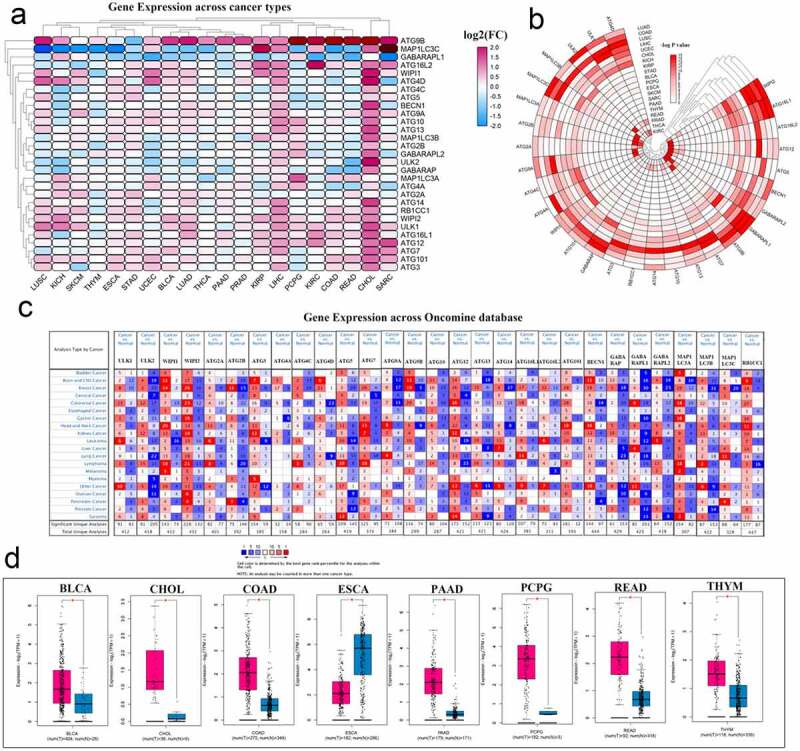
Panoramic view of the expression of autophagy-related genes in pan-cancer. (a) Gene expression across cancer types. (b) To more clearly show the difference in expression of these autophagy-related genes in tumors, a heat map of the corresponding – logP value of each gene in each tumor was constructed. The redder the color, the more intense the corresponding change in gene expression in corresponding cancer. (c) Gene expression across the oncomine database wherein red means activated and blue means suppressed. The larger the number, the darker the color, the greater the degree of change in its expression. (d) ATG9B gene expression in multiple tumors. Red represents tumor tissue and blue represents normal tissue

Modern cancer research believes that abnormal gene expression may imply that the gene may play an important role in the development of the disease. To perform a pan-cancer investigation of the mRNA expression of autophagy-related genes, we used gene expression data from the TCGA database to draw a heat map. ATG9B and ATG12 were highly expressed in various cancers. In contrast, the expression of MAP1LC3C and GABARAPL1 in multiple cancers was low. Simultaneously, most autophagy-related genes were activated in both CHOL and LIHC (Figure 2a, Table S4). To show the gene expression changes more clearly, a new heat map was generated using the negative log P-value. In this heat map, we can see a relatively high degree of changes in expression in KIRC, LIHC, and LUSC cancers. Three genes, WIPI2, ATG16L1, and GABARAPL1, showed comparable changes in various cancers (Figure 2b, Table S5). Then, we explored the expression of these genes in multiple tumors through the Oncomine database (Figure 2c). In particular, we explored the expression of ATG9B in various tumors through the tools available on the GEPIA website, and combining the TCGA and GTEx databases, and presented the data as box diagrams. ATG9B is highly expressed in most cancers (Figure 2d).

### 3.3. Correlation between autophagy-related genes and their relationship with methylation levels, cancer pathways, and drug sensitivity

**Figure 3.**
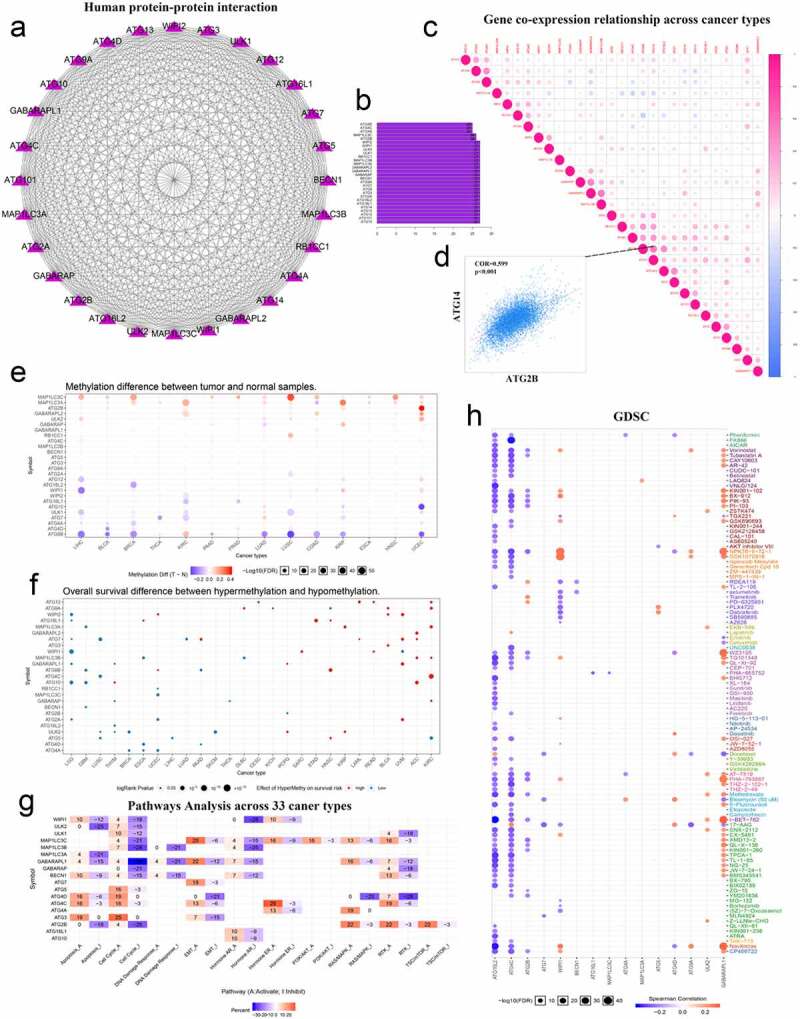
Correlation between autophagy-related genes and their relationship with methylation levels, cancer pathways, and drug sensitivity. (a) PPI networks between autophagy-related proteins. (b) Quantitative table of interactions between autophagy-related proteins. (c) Gene co-expression relationship across cancer types. Red represents a positive correlation and blue represents a negative correlation. (d) Co-expression relationship between ATG14 and ATG2B. (e) Methylation difference between tumor and normal samples. Red represents high expression and blue represents low expression. (f) Overall survival difference between hypermethylation and hypomethylation. Red represents high methylation levels as a high-risk factor, and blue represents high methylation levels as a low-risk factor. (g) Pathways analysis across 33 cancer types. Red represents activation and blue represents inhibition. (h) Sensitivity analysis of autophagy-related genes and mainstream anti-cancer drugs

Recent studies have shown that epigenetic mechanisms are essential for maintaining specific gene expression patterns and the normal development and growth of living individuals [32,33]. Among them, changes in the level of methylation can interfere with the expression and function of normal genes, thereby inducing the occurrence and development of various diseases such as cancer. To understand the potential functions of these molecules in tumors, we used the String website and Cytoscape software to map the PPI network of these genes and quantify their relationships. These molecules have a very close relationship (Figure 3a-b). The co-expression of these genes in various types of tumors was then investigated. Among them, the positive correlation between ATG14 and ATG2B was the highest, with a COR value of 0.599 (Figure 3c-d). Finally, we used the GSCALite website to investigate the methylation levels of these genes in various tumors (Figure 3e). We also explored the relationship between methylation levels and the overall survival of patients with cancer (figure 3f). We used another tool available from this website to explore the relationship between these autophagy-related genes and cancer pathways. We identified a major role of ATG4C in the hormone-ER pathway, and GABARAPL1 had a strong inhibitory effect on the cell cycle pathway (Figure 3g). We also used the integrated GDSC database from this website to investigate the sensitivity of these genes to anticancer drugs. We found that GABARAPL1 was more sensitive to navitoclax (Figure 3h).

### 3.4. Pan-cancer analysis of the hazard ratio (HR) of autophagy-related genes

**Figure 4.**
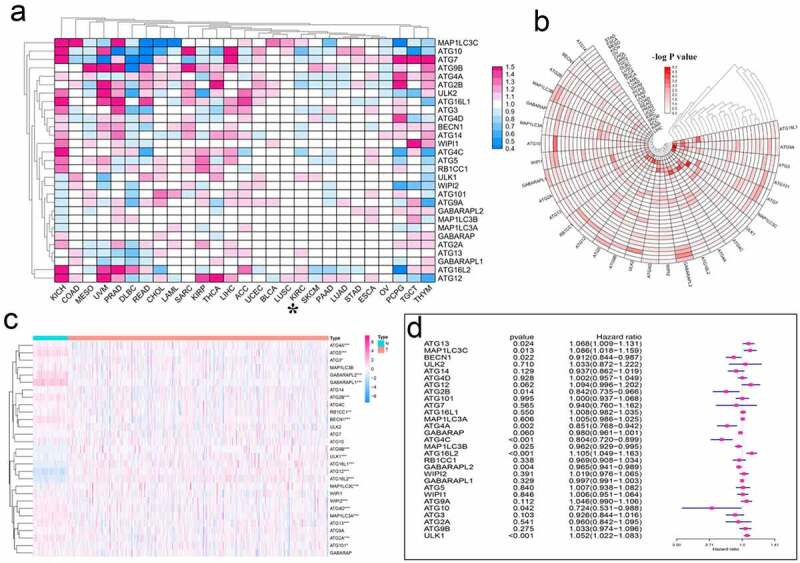
Hazard ratio analysis of autophagy-related genes in pan-cancer. (a) Risk analysis of autophagy-related genes in pan-cancer. Red represents the molecule acting as a risk factor in the corresponding tumor, and blue represents the molecule acting as a protective factor in the corresponding tumor. (b) A heat map corresponding to – logP value was drawn to more clearly show the results of risk analysis of autophagy-related genes in pan-cancer. The redder the color, the higher the credibility. (c) Expression of autophagy-related genes in KIRC patients. The redder the color, the higher the expression level, and the bluer the color, the lower the expression level. (d) Univariate Cox analysis of autophagy-related genes in KIRC patients. *P < 0.05, **P < 0.01, and ***P < 0.001

In medical and public health research, hazard ratio (HR) is often used to express the risk difference between the experimental group and the control group. To understand the effect of autophagy-related genes from a pan-cancer perspective, we analyzed the HR of these genes (Figure 4a, Table S6). To confirm the credibility of the above data, we used the logP value to draw a new heat map (Figure 4b, Table S7). To display the expression of these autophagy-related genes in KIRC in more detail, we used the KIRC gene expression data from TCGA to draw a heat map (Figure 4c). In particular, we accurately displayed the HR of autophagy-related genes in KIRC in the form of a forest map. Of these genes, ATG13, MAP1LC3C, ATG16L2, and ULK1 were potential risk factors in KIRC. In contrast, BECN1, ATG2B, ATG4A, ATG4C, MAP1LC3B, GABARAPL2, and ATG10 may be protective factors (Figure 4d, Table S8).

### 3.5. Cluster analysis of autophagy-related genes in KIRC and its clinical relevance

**Figure 5.**
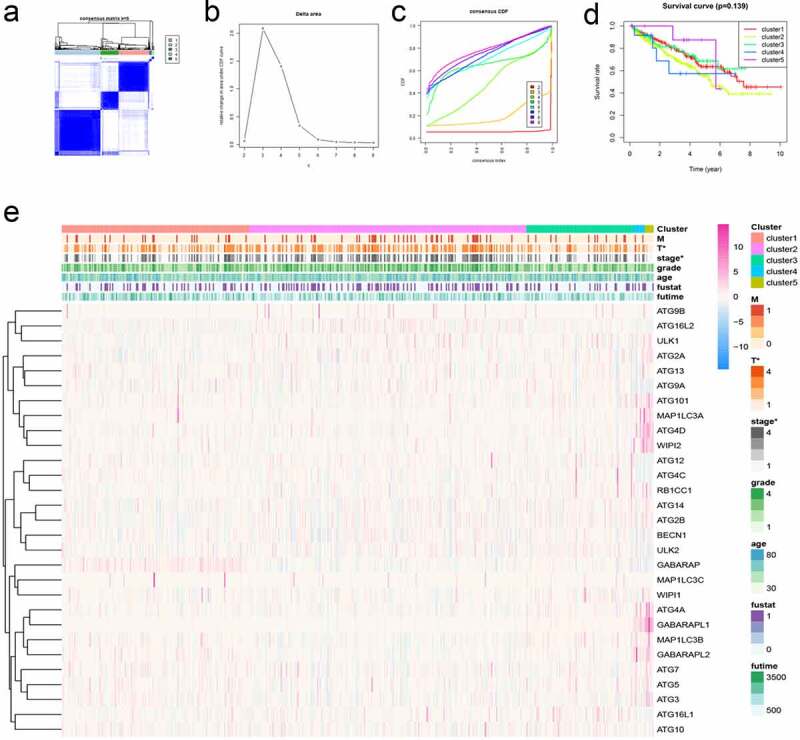
Cluster analysis of autophagy-related genes and their clinical relevance. (a) Consensus clustering matrix for k = 5. (b-c) Relative change in area under the cumulative distribution function (CDF) curve for k = 2–9. Consensus clustering CDF for k = 2–9. (d) Survival curves under different clusters. (e) Heat map between clinical features under different clusters. *P < 0.05

Cluster analysis is to group data objects based on the information found in the data describing objects and their relationships. In cancer research, cluster analysis can provide theoretical support for the precise treatment of cancer treatment. In particular, we performed cluster analysis based on the expression of these autophagy-related genes in KIRC patients. A consensus matrix was generated at k = 5, which showed a relatively good clustering effect, and the results were verified if necessary (Figure 5a-c). Then, we generated the survival curve of KIRC patients based on the results of the cluster analysis (P = 0.139) (Figure 5d). We found that there was a statistical significance between the results of the cluster analysis and the tumor stage of KIRC patients (Figure 5e).

### 3.6. Risk analysis of autophagy-related genes in KIRC and its clinical relevance

**Figure 6.**
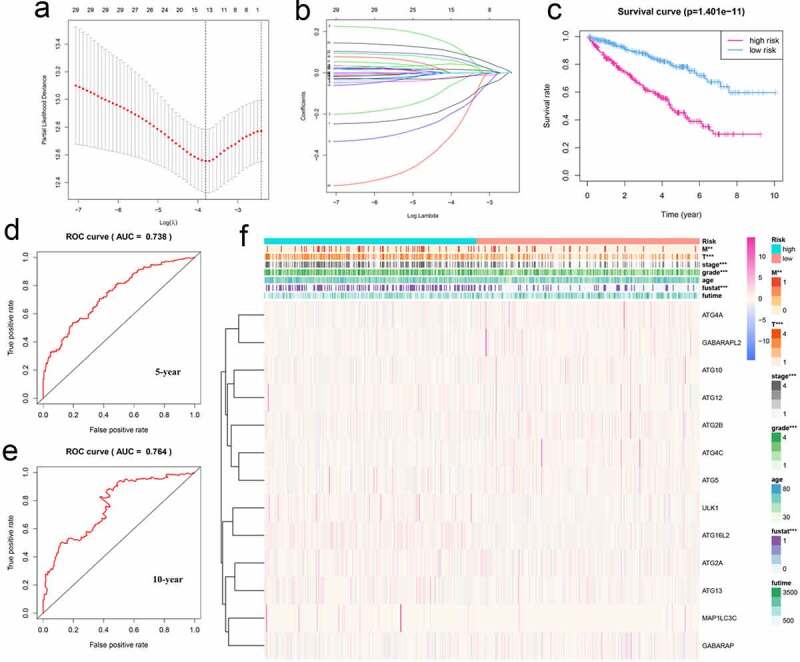
Using autophagy-related genes to establish prognostic risk model in KIRC. (a-b) Results of LASSO regression analysis and cross-validation. (c) Kaplan–Meier survival analysis between high-risk and low-risk groups according to the optimal cutoff value; (d) ROC curve for predicting 5-year survival time; (e) ROC curve for predicting 10-year survival time; (f) Heat map based on the correlation of this risk feature with clinical features. **P < 0.01, and ***P < 0.001

**Figure 7.**
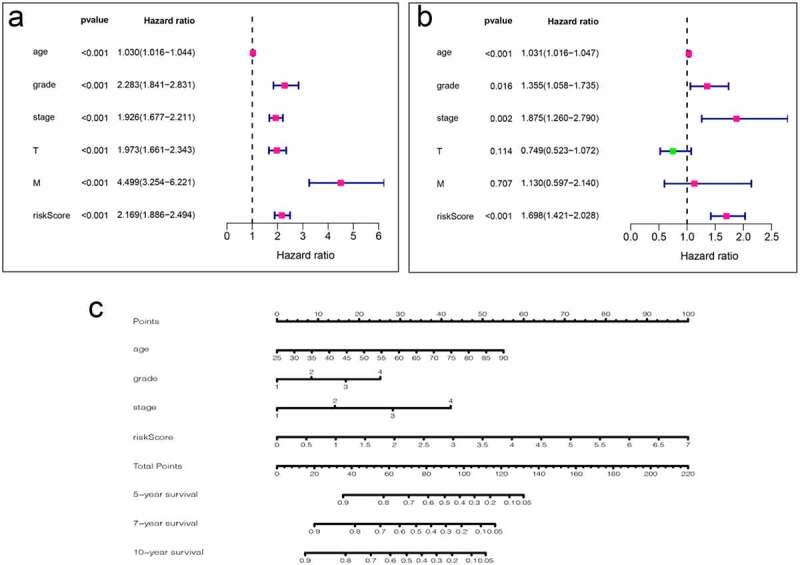
Analysis of clinical relevance of risk models. (a) Univariate Cox analysis. (b) Multivariate Cox analysis. (c) A new nomogram was drawn based on this prognostic risk signature. The value of each variable gets a score on the dot scale axis. The total score can be easily calculated by adding up each score and projecting the total score to a lower total score system. We can estimate the risk for predicting 5-, 7 – or 10-year survival in KIRC

To use these autophagy-related genes to build a risk model for KIRC, we first performed a LASSO regression analysis on these genes and verified their usability (Figure 6a-b). We derived a risk model consisting of 13 genes, including ATG4A, GABARAPL2, ATG10, ATG12, ATG2B, ATG4C, ATG5, ULK1, ATG16L2, ATG2A, ATG13, MAP1LC3C, and GABARAP. Based on the expression of these genes, we divided KIRC patients into high-risk and low-risk groups and plotted survival curves (P = 1.401e-11) (Figure 6c). We then plotted the ROC curve for this risk model and found that the ROC value for 5 years was 0.738 and that for 10 years was 0.764 (Figure 6d-e), indicating that this risk model is highly accurate. We then combined this risk model with the clinical characteristics of KIRC patients and displayed them in the form of a heat map. We found that the risk model has strong statistical significance with the five clinical features including metastasis, tumor, stage, grade, and fustat in KIRC patients (figure 6f). Finally, we performed univariate Cox analysis and multivariate Cox analysis based on this model (Figure 7a-b, Table S9, S10). We found that age, grade, stage, and risk score are independent risk factors for KIRC patients. Based on this risk signature, a nomogram that can predict the risks of KIRC patients in 5-, 7- and 10-year is drawn (Figure 7c). The value of each variable gets a score on the points scale axis. The nomogram generates a total of nine rows. The second, third, fourth and fifth rows represent age, grade, stage and riskScore respectively. The sixth row’s total points are accumulated from each score assigned to age, grade, stage, and riskScore. We can easily estimate the 5-, 7- and 10-year survival rates of KIRC patients from the total points.

## 4. Discussion

Under normal physiological conditions, autophagy can remove damaged organelles and proteins from the cells to maintain cell homeostasis. Under abnormal pathological conditions, autophagy is related to the occurrence and development of Parkinson’s disease, malignant tumors, and other diseases. Autophagy plays a dual role in the appearance and development of malignant tumors [6,34]. In the early growth of malignant tumors, autophagy can inhibit the continuous growth of precancerous cells, thereby inhibiting the growth of malignant tumors[35]. In the later stages of the development of malignant tumors, malignant tumor cells are in a state of hypoxia and nutritional deficiency [34,36]. The clinical trials that have been carried out have demonstrated the feasibility and potential benefits of inhibiting autophagy in a variety of cancer models, including glioblastoma, pancreatic cancer, melanoma, sarcoma, and multiple myeloma [37–42]. Autophagy provides energy for the growth of tumors by degrading proteins, organelles, and macromolecular substances in the cells, thereby promoting the development of malignant tumors[43].

KIRC is a disease of the renal parenchymal urinary tubule epithelial system, and its pathogenesis is very complicated. Tumor cells begin to grow from within the kidney parenchyma. As the disease progresses, the tumor volume gradually becomes larger, and tumor cells start to compress and infiltrate the surrounding renal pelvis and calyx, thereby destroying the extrarenal capsule area [44]. In most cases, KIRC is relatively resistant to radiotherapy and chemotherapy, and surgery is the primary treatment. Despite the early surgical treatment, 30% of tumor patients eventually metastasize [45]. To improve the prognosis of kidney cancer patients, we comprehensively used bioinformatics to determine the expression, mutation, and overall survival of autophagy-related genes in pan-cancer, and to establish a model that is closely related to the prognosis of KIRC patients.

In our study, we used a large number of bioinformatics-related tools to conduct an in-depth exploration of the pan-cancer’s autophagy-related genes. For the first time, the expression, variation, and overall survival of these genes in pan-cancer have been displayed in the form of a heat map, which provides many potential research directions for future research on autophagy-related cancer. Since we focused on KIRC, we conducted more in-depth research on KIRC. Cluster analysis and risk analysis were performed on KIRC patients. In cluster analysis, we divided KIRC patients into five clusters and found a clear correlation between them and the two clinical features including tumor type and stage. In the risk analysis, we created a risk model among KIRC patients. This risk model contains 13 genes, including ATG4A, GABARAPL2, ATG10, ATG12, ATG2B, ATG4C, ATG5, ULK1, ATG16L2, ATG2A, ATG13, MAP1LC3C, and GABARAP. We divided KIRC patients into high- and low-risk groups, and found that the prognosis of patients in the high-risk group is significantly worse than that of the low-risk group. We found that there is a correlation between this risk model and the patient’s five clinical characteristics including metastasis, tumor, stage, grade, and fustat. The AUC value of the 5-year ROC curve of this risk model is 0.738, and the AUC value of the 10-year ROC curve is 0.764. Both of them exceed 0.7, indicating that this risk model is very accurate and reliable.

Next, we conducted an in-depth analysis of the role of these 13 autophagy-related genes as potential prognostic targets in cancer. The function of ATG2 was unknown for a long time after it was discovered. However, in a recent study, Valverde et al. reported that it is likely to be a lipid transfer protein, which plays a role in maintaining the lipid homeostasis of autophagosomes, supporting the biological behavior of organelles [46]. ATG2A/B mutation loss during biological evolution can induce non-classical caspase-8 activation and apoptosis [47]. ATG2A has been reported as a target of miRNA-541, and its imbalance in hepatocellular carcinoma plays a crucial role in patients’ response to sorafenib treatment. Modulating ATG2A expression can prolong the overall survival of patients with hepatocellular carcinoma by eliminating drug resistance [48]. ATG4 protease is a cysteine protease that plays an essential role in the lipidation and delipidation of LC3 during autophagy. In oncology, multiple isoforms of Atg4 protease have been identified as potential targets for cancer treatment [49]. ATG4A is associated with the clinical stage and progression-free survival in patients with ovarian cancer [50]. Moreover, ATG4A is more highly expressed in gastric cancer tissues than in normal tissues and it can promote the EMT of gastric cancer cells [51]. Other researchers have found that ATG4A is associated with drug resistance and stemness in tumors [52,53]. Another isoform molecule of ATG4, ATG4C, has also been extensively studied in the field of cancer. Recently, researchers have found that ATG4C is highly expressed in glioblastomas, and its expression increased with the number of gliomas. Knockdown of ATG4C expression in gliomas can induce cell cycle arrest, thereby inhibiting cancer cell proliferation [54]. Additionally, in breast cancer and hepatocellular carcinoma, silencing the ATG4C gene can inhibit the occurrence of autophagy, thus affecting tumor development [55,56]. All the above findings suggest that the two isoforms of ATG4 play an essential role in tumorigenesis and cancer treatment. The development of specific inhibitors of ATG4A and ATG4C may lead to new approaches for tumor treatment in the future [57].

Some researchers have found that down-regulation of ATG5 expression under hypoxic conditions can inhibit the expression of EMT markers N-cadherin and vimentin, thereby inducing malignant development of prostate cancer cells [58]. Besides, ATG5 can be used as a target of lncRNA-ATB to affect the development of hepatocellular carcinoma by affecting autophagy [59]. ATG10 is an E2-like enzyme involved in Ub-like modification, playing an essential role in the formation of autophagosomes. ATG10 expression is considered to be closely related to lymphatic infiltration and lymph node metastasis of colorectal cancer, and lymphatic infiltration and lymph node metastasis can affect the overall survival of patients [60–62]. However, in gastric cancer, the low expression of ATG10 affects its lymph node metastasis [63]. This indicates that ATG10 is expressed differently in different types of tumors and plays various roles. The specific mechanism requires more in-depth study.

Similarly, many studies have shown that regulating the expression level of ATG12 can increase tumor cells’ sensitivity to anti-cancer drugs and significantly improve the effectiveness of tumor treatment [64–66]. Some researchers have used the expression level of ATG16L2 to detect the response of cells to cisplatin, speculating that it may be a biomarker for tumor cells resistant to platinum-based drugs [67]. Besides, the abnormal activation of ULK1 in non-small cell lung cancer can affect the skeletal dynamics of cancer cells and release related cell movement effectors, leading to distant metastasis [68]. The interaction between ATG13 and ULK1 is critical in the biological process of autophagy [69]. MAP1LC3C can mediate the selective autophagy process of METRTK, thereby inhibiting the invasion of cancer cells, and may play an essential role in the distant metastasis of cancer cells [70]. Some researchers have found that GABARAP is abnormally activated in colorectal cancer tissue, and its overexpression is positively correlated with the malignancy of the tumor, affecting the patient’s overall survival [71]. We believe there will be more and more drugs targeting autophagy-related genes [72]. It can be seen that the 13 target genes play various roles in the development of multiple tumors. Therefore, these target genes are promising targets for cancer treatment in the future.

## 5. Conclusions

In conclusion, in this study, through a series of rigorous analyses, we used 13 genes of the autophagy-related genes to construct a new prognostic model for KIRC. The AUC value of the 5-year ROC curve of this model is 0.738, indicating that it can accurately predict the prognosis of KIRC patients and is expected to assist doctors in clinical diagnosis, decision-making, and monitoring. However, the potential molecular mechanism of these 13 genes in KIRC requires further study. It is undeniable that there are still many shortcomings in this study. This study only uses public databases to explore the underlying mechanisms of autophagy-related genes in cancer, and has not been verified by single-center or multi-center clinical data. Therefore, in the future, we will continue to explore the potential biological role of these key autophagy-related genes in KIRC. We also believe that our research could provide reliable data for future scientific research on autophagy.

## Supplementary Material

Supplemental Material

## Data Availability

The data used to support the findings of this study are available from the corresponding author upon request.

## Abbreviations

KIRC: Kidney renal clear cell carcinoma; KIRP: Kidney renal papillary cell carcinoma; KICH: Kidney chromophobe; CNV: Copy number variation; TCGA: The cancer genome atlas; SNV: Single nucleotide variation; LASSO: Least absolute shrinkage and selection operator; ATP: Adenosine triphosphate; RCC: Renal cell carcinoma; ATG4A: Autophagy Related 4A Cysteine Peptidase; GABARAPL2: GABA Type A Receptor Associated Protein Like 2; ATG10: Autophagy Related 10; ATG12: Autophagy Related 12; ATG2B: Autophagy Related 2B; ATG4C: Autophagy Related 4C; ATG5: Autophagy Related 5; ULK1: Unc-51 Like Autophagy Activating Kinase 1; ATG16L2: Autophagy Related 16 Like 2; ATG2A: Autophagy Related 2A; ATG13: Autophagy Related 13; MAP1LC3C: Microtubule Associated Protein 1 Light Chain 3 Gamma; GABARAP: GABA Type A Receptor-Associated Protein; TCGA: The Cancer Genome Atlas; NCI: National Cancer Institute; NHGRI: National Human Genome Research Institute; ATG9B: Autophagy Related 9B; GDSC: Genomics of Drug Sensitivity in Cancer; CTRP: Clinical Trials Reporting Program; CRAN: Comprehensive R Archive Network; ATG16L2: Autophagy Related 16 Like 2; BECN1: Beclin 1; ACC: Adrenocortical carcinoma; OV: Ovarian serous cystadenocarcinoma; WIPI2: WD Repeat Domain, Phosphoinositide Interacting 2; MAP1LC3A: Microtubule Associated Protein 1 Light Chain 3 Alpha; RB1CC1: RB1 Inducible Coiled-Coil 1; UVM: Uveal Melanoma; UCS: Uterine Carcinosarcoma; ATG7: Autophagy Related 7; ULK2: Unc-51 Like Autophagy Activating Kinase 2; UCEC: Uterine Corpus Endometrial Carcinoma; STAD: Stomach adenocarcinoma; COAD: Colon adenocarcinoma; SKCM: Skin Cutaneous Melanoma; ATG2B: Autophagy Related 2B; GABARAPL1: GABA Type A Receptor Associated Protein Like 1; GEPIA: Gene Expression Profiling Interactive Analysis; MAP1LC3B: Microtubule Associated Protein 1 Light Chain 3 Beta.

## Research Highlights:

(1) This study shows the variation of autophagy-related genes in pan-cancer.

(2) Use cluster analysis to successfully divide KIRC patients into five clusters.

(3) Use LASSO regression analysis to build a prognostic risk model in KIRC.

(4) Based on the risk model, a prognostic-related Nomo diagram was drawn.
