# Supplementary material for: A new prognostic risk model based on autophagy-related genes in kidney renal clear cell carcinoma
Source: Bioengineered. 2021 Oct 12;12(1):7805–19. doi: 10.1080/21655979.2021.1976050 (PMC8806698; doi:10.1080/21655979.2021.1976050)
Supplement: Supplemental Material [file KBIE_A_1976050_SM3653.pdf]

Table S1: The CNV gain frequency of autophagy-related genes in pan-cancer

	ACC	BLCA	BRCA	CESC	CHOL	COAD	DLBC	ESCA
ULK1	0.6	0.057971	0.043112	0.053061	0.060606	0.076152	0.034483	0.072917
ULK2	0.044444	0.062802	0.054358	0.020408	0.030303	0.02004	0.034483	0.03125
ATG2A	0.011111	0.045894	0.039363	0.044898	0	0.016032	0.034483	0.0625
ATG2B	0.155556	0.028986	0.032802	0.110204	0	0.008016	0.034483	0.125
ATG3	0.077778	0.161836	0.078725	0.485714	0.030303	0.0501	0.172414	0.427083
ATG4A	0.011111	0.033816	0.015933	0.02449	0	0.018036	0.034483	0.125
ATG4C	0.011111	0.038647	0.048735	0.15102	0.181818	0.002004	0	0.020833
ATG4D	0.477778	0.048309	0.053421	0.057143	0	0.038076	0	0.020833
ATG5	0.1	0.024155	0.086223	0.028571	0.030303	0.026052	0	0.03125
BECN1	0.044444	0.082126	0.088097	0.044898	0.060606	0.076152	0.034483	0.052083
ATG7	0.055556	0.23913	0.058107	0.036735	0	0.018036	0.103448	0
GABARAPL	0.611111	0.089372	0.089972	0.04898	0.090909	0.092184	0.034483	0.21875
GABARAPL	0.366667	0.077295	0.034677	0.073469	0.030303	0.078156	0.034483	0.0625
MAP1LC3A	0.422222	0.285024	0.172446	0.265306	0.090909	0.649299	0	0.239583
MAP1LC3C	0.088889	0.108696	0.56701	0.285714	0.454545	0.112224	0.137931	0.270833
MAP1LC3B	0.377778	0.05314	0.031865	0.085714	0.030303	0.078156	0.034483	0.083333
GABARAP	0.033333	0.012077	0.007498	0	0	0.008016	0.034483	0.052083
ATG9A	0.066667	0	0.020619	0.008163	0.060606	0.028056	0	0
ATG9B	0.388889	0.077295	0.08716	0.04898	0.060606	0.278557	0.034483	0.072917
ATG10	0.555556	0.009662	0.041237	0.02449	0.060606	0.012024	0.034483	0.020833
ATG12	0.555556	0.014493	0.045923	0.032653	0.151515	0.012024	0.068966	0
ATG13	0.022222	0.028986	0.059044	0.028571	0	0.024048	0.068966	0.052083
ATG14	0.144444	0.028986	0.048735	0.102041	0.030303	0.016032	0.068966	0.15625
ATG16L1	0.055556	0	0.008435	0	0.090909	0.03006	0	0.010417
ATG16L2	0.044444	0.115942	0.125586	0.053061	0.030303	0.018036	0.068966	0.28125
RB1CC1	0.322222	0.229469	0.344892	0.126531	0.060606	0.300601	0.068966	0.229167
WIPI1	0.077778	0.183575	0.258669	0.122449	0.151515	0.112224	0.068966	0.072917
WIPI2	0.411111	0.147343	0.119025	0.077551	0.090909	0.400802	0.172414	0.260417
ATG101	0.633333	0.048309	0.045923	0.04898	0.090909	0.098196	0.034483	0.020833

GBM	HNSC	KICH	KIRC	KIRP	LGG	LIHC	LUAD	LUSC
0.030255	0.017241	0.333333	0.047538	0.299342	0.016886	0.044855	0.055985	0.041985
0.007962	0.060345	0	0.013582	0.447368	0.007505	0.055409	0.069498	0.066794
0.012739	0.094828	0.348485	0.008489	0.026316	0.026266	0.010554	0.054054	0.038168
0.012739	0.12069	0.454545	0.011885	0.013158	0.005629	0.013193	0.063707	0.083969
0.035032	0.284483	0.287879	0.018676	0.233553	0.003752	0.026385	0.063707	0.377863
0.004777	0.025862	0	0.010187	0.006579	0.011257	0.058047	0.027027	0.057252
0.06051	0	0	0.005093	0.006579	0.016886	0.042216	0.044402	0.024809
0.299363	0.017241	0.318182	0.016978	0.009868	0.056285	0.029024	0.007722	0.049618
0.011146	0.025862	0.030303	0.003396	0.009868	0.001876	0.036939	0.005792	0.043893
0.015924	0.034483	0	0.018676	0.490132	0.011257	0.113456	0.096525	0.057252
0.038217	0.008621	0.333333	0.006791	0.174342	0.020638	0.050132	0.005792	0.015267
0.063694	0.198276	0.454545	0.052632	0.319079	0.050657	0.021108	0.084942	0.183206
0.012739	0.060345	0.257576	0.037351	0.384868	0.011257	0.005277	0.061776	0.038168
0.280255	0.086207	0.409091	0.047538	0.259868	0.041276	0.116095	0.125483	0.196565
0.078025	0.155172	0.015152	0.044143	0.039474	0.037523	0.559367	0.30888	0.192748
0.017516	0.043103	0.348485	0.040747	0.378289	0.009381	0.013193	0.034749	0.024809
0.003185	0.043103	0	0.013582	0.434211	0.003752	0.007916	0.001931	0.007634
0.012739	0	0.015152	0.035654	0.042763	0.007505	0.029024	0.019305	0.009542
0.703822	0.094828	0.515152	0.157895	0.542763	0.262664	0.176781	0.146718	0.114504
0.019108	0.008621	0.318182	0.144312	0.069079	0.013133	0.100264	0.042471	0
0.012739	0	0.272727	0.303905	0.085526	0.013133	0.137203	0.028958	0.001908
0.009554	0.051724	0.333333	0.011885	0.016447	0.020638	0.021108	0.054054	0.041985
0.004777	0.103448	0.424242	0.008489	0	0.009381	0.01847	0.092664	0.061069
0.011146	0.008621	0.015152	0.035654	0.046053	0.003752	0.023747	0.030888	0.013359
0.017516	0.12069	0.424242	0.008489	0.029605	0.045028	0.036939	0.102317	0.101145
0.033439	0.267241	0.287879	0.035654	0.065789	0.041276	0.350923	0.220077	0.187023
0.028662	0.094828	0	0.028862	0.539474	0.052533	0.218997	0.162162	0.167939
0.69586	0.224138	0.378788	0.147708	0.516447	0.157598	0.182058	0.281853	0.177481
0.05414	0.043103	0.454545	0.04584	0.282895	0.003752	0.023747	0.07722	0.041985

MESO	OV	PAAD	PCPG	PRAD	READ	SARC	SKCM	STAD
0.05814	0.119237	0	0.066667	0.010142	0.060241	0.05	0.021186	0.033937
0	0.014308	0.04698	0	0.01217	0.012048	0.366667	0.016949	0.013575
0.05814	0.147854	0	0.013333	0.020284	0.036145	0.116667	0.023305	0.038462
0.011628	0.101749	0.020134	0.006667	0.008114	0.012048	0.15	0.027542	0.015837
0.05814	0.408585	0.006711	0	0.016227	0.060241	0.116667	0.036017	0.063348
0.011628	0.08903	0.006711	0	0	0.012048	0.041667	0.006356	0.020362
0.034884	0.208267	0	0.013333	0	0	0.258333	0.080508	0.0181
0.081395	0.27345	0	0.06	0.004057	0.036145	0.241667	0.027542	0.024887
0	0.101749	0.006711	0.013333	0	0.096386	0.125	0.010593	0.052036
0.069767	0.015898	0.013423	0.02	0.002028	0.108434	0.033333	0.036017	0.049774
0.034884	0.117647	0.006711	0.013333	0.010142	0.036145	0.141667	0.03178	0.0181
0.023256	0.384738	0.033557	0.02	0.002028	0.144578	0.125	0.07839	0.074661
0.023256	0.041335	0	0.02	0.004057	0.120482	0.033333	0.033898	0.020362
0.034884	0.419714	0.040268	0.013333	0.018256	0.86747	0.191667	0.211864	0.321267
0.093023	0.383148	0.154362	0.086667	0.024341	0.144578	0.05	0.330508	0.11086
0.023256	0.034976	0.006711	0.02	0	0.144578	0.033333	0.023305	0.024887
0	0.058824	0.006711	0	0	0	0.066667	0.010593	0.004525
0.011628	0.158983	0.033557	0.006667	0.006085	0.048193	0.033333	0.021186	0.031674
0.069767	0.418124	0.020134	0.06	0.054767	0.337349	0.125	0.389831	0.079186
0.046512	0.017488	0	0.033333	0.006085	0.060241	0.116667	0.050847	0.015837
0.05814	0.074722	0	0.026667	0.008114	0.060241	0.108333	0.038136	0.022624
0.011628	0.106518	0.013423	0.013333	0.004057	0.024096	0.116667	0.036017	0.042986
0.011628	0.063593	0.020134	0.02	0.008114	0.036145	0.166667	0.038136	0.024887
0.011628	0.149444	0.033557	0.006667	0.002028	0.048193	0.025	0.012712	0.015837
0.05814	0.27504	0.033557	0.013333	0.020284	0.036145	0.116667	0.069915	0.085973
0.069767	0.354531	0.053691	0.04	0.127789	0.39759	0.2	0.25	0.214932
0.151163	0.160572	0.026846	0.04	0.010142	0.120482	0.158333	0.158898	0.065611
0.127907	0.136725	0.067114	0.106667	0.064909	0.46988	0.3	0.319915	0.217195
0.023256	0.209857	0	0.04	0.016227	0.072289	0.033333	0.021186	0.033937

TGCT	THCA	THYM	UCEC	UCS	UVM
0.102564	0.011742	0	0.041894	0.017857	0
0.019231	0.005871	0.033333	0.023679	0.142857	0.0375
0	0	0	0.040073	0.142857	0.0875
0.096154	0.005871	0	0.051002	0.017857	0.0625
0.038462	0	0	0.083789	0.214286	0
0.012821	0.001957	0	0.020036	0.071429	0
0.057692	0	0	0.040073	0.107143	0
0.019231	0	0	0.120219	0.160714	0.0125
0.012821	0	0	0.051002	0.232143	0.05
0.064103	0.007828	0.066667	0.023679	0.178571	0.05
0.019231	0	0	0.061931	0.071429	0
0.884615	0.005871	0.033333	0.052823	0.178571	0
0.012821	0.005871	0	0.01275	0	0
0.032051	0.003914	0.033333	0.143898	0.392857	0.0875
0.096154	0.052838	0.066667	0.307832	0.446429	0.15
0.00641	0.005871	0	0.010929	0	0
0.019231	0.003914	0	0.027322	0.089286	0.0625
0.044872	0.001957	0	0.058288	0.160714	0.05
0.269231	0.035225	0.033333	0.087432	0.196429	0.075
0	0.013699	0	0.009107	0.035714	0
0	0.015656	0	0.01275	0.053571	0
0.012821	0	0	0.03643	0.017857	0.075
0.083333	0.001957	0	0.034608	0.017857	0.05
0.00641	0.001957	0	0.052823	0.178571	0.025
0	0	0	0.043716	0.125	0.0875
0.397436	0	0	0.224044	0.5	0.5875
0.064103	0.007828	0.066667	0.071038	0.321429	0.1875
0.397436	0.021526	0	0.089253	0.232143	0.0875
0.166667	0.009785	0	0.054645	0.196429	0

Table S2: The CNV loss frequency of autophagy-related genes in pan-cancer

	ACC	BLCA	BRCA	CESC	CHOL	COAD	DLBC	ESCA
ULK1	0.02222	0.04831	0.0478	0.00816	0.09091	0.01403	0	0.03125
ULK2	0.14444	0.16425	0.19213	0.13061	0.09091	0.31062	0.03448	0.08333
ATG2A	0.14444	0.01691	0.06467	0.01224	0	0.03808	0	0.02083
ATG2B	0.05556	0.07488	0.09747	0.06122	0.42424	0.20441	0	0.05208
ATG3	0.1	0.01208	0.01781	0.02449	0.09091	0.01002	0	0.03125
ATG4A	0.03333	0.01691	0.01031	0	0	0.00401	0	0
ATG4C	0.23333	0.01691	0.07591	0.01224	0.15152	0.07415	0.03448	0.08333
ATG4D	0.02222	0.05072	0.03187	0.07347	0	0.01603	0	0.03125
ATG5	0.11111	0.11836	0.14433	0.13061	0.45455	0.03808	0.27586	0.08333
BECN1	0.17778	0.00966	0.10965	0.02041	0	0.02806	0	0
ATG7	0.14444	0.02899	0.03655	0.27755	0.51515	0.0481	0	0.375
GABARAPL	0.02222	0.04106	0.05436	0.07755	0.0303	0.02405	0.03448	0.01042
GABARAPL	0.06667	0.08213	0.33739	0.05306	0.09091	0.01403	0.03448	0.02083
MAP1LC3/	0.01111	0.00242	0.00937	0.00408	0	0	0	0.01042
MAP1LC3(	0.11111	0.00725	0.01031	0	0	0.01002	0.2069	0.02083
MAP1LC3I	0.06667	0.09662	0.35614	0.05306	0.09091	0.02806	0.03448	0.03125
GABARAP	0.2	0.20773	0.27741	0.2	0.12121	0.36673	0.03448	0.0625
ATG9A	0.05556	0.17633	0.04217	0.22449	0.0303	0.00601	0.03448	0.09375
ATG9B	0.06667	0.02174	0.05998	0.1102	0.06061	0.01002	0.03448	0.10417
ATG10	0.01111	0.13527	0.1059	0.1102	0.0303	0.06413	0	0.20833
ATG12	0.01111	0.11353	0.07873	0.10612	0	0.10621	0.06897	0.16667
ATG13	0.16667	0.14734	0.04967	0.10204	0.06061	0.01403	0	0.03125
ATG14	0.06667	0.07005	0.07404	0.01633	0.27273	0.17635	0	0.05208
ATG16L1	0.06667	0.21981	0.05998	0.2898	0	0.02405	0	0.125
ATG16L2	0.14444	0.03382	0.09372	0.13469	0.06061	0.03006	0	0.13542
RB1CC1	0.06667	0.00725	0.02718	0.00816	0.0303	0.02605	0.03448	0.03125
WIPI1	0.17778	0.00242	0.02343	0.01633	0	0.03006	0	0.01042
WIPI2	0.03333	0.01691	0.03561	0.0449	0.0303	0	0.03448	0.02083
ATG101	0	0.02174	0.03187	0.00408	0	0	0	0.02083

GBM	HNSC	KICH	KIRC	KIRP	LGG	LIHC	LUAD	LUSC
0.02548	0.03448	0	0.0034	0.01316	0.01876	0.05805	0.02124	0.01527
0.04936	0.07759	0.59091	0.02207	0.03289	0.0075	0.22691	0.08108	0.10687
0.04618	0.01724	0.06061	0.0034	0.00658	0.01501	0.04222	0.01351	0.01527
0.17516	0.06897	0.04545	0.13073	0.10855	0.11632	0.16887	0.05212	0.04389
0.03981	0.02586	0.0303	0.10866	0.01316	0.03565	0.00792	0.01737	0.02099
0.00478	0	0	0.00509	0	0.00188	0.00264	0.00579	0.00763
0.00955	0.05172	0.66667	0.05433	0.07566	0.32458	0.09235	0.02896	0.05153
0.00955	0.00862	0	0.0034	0.01645	0.00188	0.08707	0.09846	0.04962
0.12739	0.02586	0.65152	0.08319	0.05263	0.06942	0.20053	0.14672	0.03435
0.0207	0	0.54545	0.01019	0.00329	0.01313	0.01847	0.00579	0.02481
0.02389	0.27586	0.04545	0.43124	0.03289	0.01689	0.02902	0.05792	0.20229
0.06051	0.01724	0	0.0017	0.00329	0.01313	0.1029	0.06371	0.00191
0.07643	0.06897	0.06061	0.01188	0.01316	0.01126	0.24274	0.03668	0.07634
0.00478	0.00862	0	0	0	0	0	0.01931	0.00763
0.02548	0	0.69697	0.04244	0.03618	0.01689	0.00792	0.00386	0.00763
0.07166	0.08621	0.06061	0.01188	0.02961	0.01689	0.26385	0.08301	0.09924
0.07325	0.10345	0.68182	0.02716	0.04934	0.00938	0.44591	0.11776	0.12595
0.00159	0.11207	0.39394	0.03056	0.02961	0.01501	0.02639	0.00965	0.06679
0.00159	0.10345	0	0.0034	0.00658	0.00188	0.03958	0.00965	0.03435
0.01274	0.16379	0.09091	0.00679	0.00658	0.01126	0.0343	0.05405	0.16031
0.01592	0.16379	0.09091	0.0034	0.00987	0.01501	0.02639	0.05985	0.14885
0.07166	0.06034	0.09091	0.00509	0.02961	0.07317	0.01319	0.01931	0.03053
0.18312	0.06034	0.01515	0.13243	0.09211	0.07692	0.12929	0.03668	0.0458
0.04459	0.10345	0.36364	0.05093	0.04276	0.0788	0.03694	0.00965	0.07252
0.04777	0.11207	0.09091	0.00679	0.01645	0.01126	0.04222	0.00579	0.02863
0.01752	0.01724	0.15152	0.04244	0.02303	0.01126	0.05013	0.00965	0.02099
0.00478	0	0.66667	0.01358	0.00329	0.00375	0.00792	0.00193	0.00382
0.00637	0.02586	0	0.0017	0	0.01501	0.00792	0.02317	0.0229
0.05255	0	0	0	0	0.0394	0.00792	0.01158	0.00573

MESO	OV	PAAD	PCPG	PRAD	READ	SARC	SKCM	STAD
0.03488	0.20827	0.03356	0.03333	0.03448	0.0241	0.04167	0.08898	0.03394
0.09302	0.56916	0.10067	0.26667	0.03245	0.44578	0.05833	0.13983	0.10181
0	0.0318	0.00671	0.12	0	0.03614	0.08333	0.14407	0.01357
0.2093	0.21622	0.02013	0.06667	0.02028	0.20482	0.05833	0.13559	0.0362
0.01163	0.03339	0.01342	0.43333	0.0142	0	0.09167	0.0678	0.00679
0.01163	0.0318	0.00671	0	0.00203	0	0.13333	0.01271	0.00905
0.03488	0.0779	0.04698	0.62667	0.03854	0.10843	0.05833	0.04237	0.01357
0	0.13514	0.01342	0	0.01014	0.0241	0.025	0.03178	0.03846
0.18605	0.34022	0.12081	0.14	0.18661	0.07229	0.15	0.33475	0.04525
0.03488	0.43402	0.01342	0.16667	0.04665	0.07229	0.06667	0.05932	0.00905
0.05814	0.1558	0.03356	0.21333	0.03651	0.10843	0.075	0.0678	0.03846
0.03488	0.07949	0.03356	0.02	0.10142	0.08434	0.13333	0.03178	0.05204
0.04651	0.52305	0.02013	0.01333	0.20081	0.0241	0.16667	0.13347	0.04072
0.01163	0.02067	0	0.01333	0.01826	0	0.04167	0.01059	0.00679
0.03488	0.0461	0	0.08	0.01826	0.0241	0.13333	0.01695	0.00905
0.10465	0.52146	0.02013	0.02	0.22515	0.03614	0.19167	0.1822	0.05882
0.12791	0.44992	0.12752	0.33333	0.17444	0.49398	0.29167	0.15466	0.09729
0.04651	0.0938	0.00671	0.01333	0.01014	0	0.11667	0.03602	0.00679
0	0.03975	0.01342	0.06	0.01623	0.01205	0.175	0.01907	0.05204
0.02326	0.4849	0.04027	0.04	0.0568	0.16867	0.03333	0.09534	0.09729
0.04651	0.32432	0.01342	0.04	0.06694	0.16867	0.04167	0.11229	0.09729
0	0.10811	0.01342	0.24	0.0426	0.03614	0.05	0.12924	0.02941
0.13953	0.22258	0.00671	0.06	0.00811	0.15663	0.04167	0.11017	0.02036
0.03488	0.11606	0.00671	0.02	0.01826	0	0.26667	0.08475	0.02036
0	0.03498	0	0.15333	0.00203	0.03614	0.04167	0.11441	0.01584
0.03488	0.02067	0.01342	0.06	0.02637	0.01205	0.075	0.02331	0.01584
0.01163	0.19078	0.0604	0.04667	0.01014	0.0241	0.025	0.02966	0.02036
0	0.25119	0	0	0.01014	0	0.05833	0.00847	0.00679
0.02326	0.05882	0.02013	0.01333	0.00406	0	0.075	0.07203	0.02036

TGCT	THCA	THYM	UCEC	UCS	UVM
0	0	0	0.04554	0.19643	0.025
0.01282	0.00587	0	0.12386	0.39286	0.0375
0.08974	0.00978	0	0.02368	0.125	0
0.00641	0.00196	0	0.03825	0.14286	0.025
0	0	0	0.01275	0.10714	0.5125
0	0	0	0.00182	0.03571	0.0125
0.01923	0.00391	0	0.01093	0.01786	0.2875
0.01282	0.01174	0	0.04918	0.25	0.025
0.01282	0.00587	0.06667	0.02186	0.05357	0.35
0.00641	0	0	0.12204	0.23214	0.05
0	0.00978	0.06667	0.05282	0.21429	0.5
0	0.00196	0	0.02914	0.14286	0.0125
0.02564	0.00587	0.06667	0.20036	0.39286	0.25
0	0	0	0.00546	0	0
0	0	0	0.00546	0	0.0125
0.03846	0.00587	0.06667	0.21129	0.375	0.25
0.05128	0.01174	0.06667	0.14936	0.44643	0.0375
0	0.00587	0	0.02004	0	0.0125
0.00641	0	0	0.04736	0.125	0.0125
0.02564	0	0	0.09654	0.23214	0.05
0.03205	0	0	0.06375	0.10714	0.0625
0.04487	0.00587	0	0.051	0.25	0.025
0	0	0.03333	0.04736	0.19643	0.025
0.01282	0.00587	0	0.02186	0.01786	0.05
0.08974	0.00978	0	0.03097	0.19643	0.0125
0.00641	0	0	0.00546	0.07143	0
0	0	0	0.05829	0.03571	0
0	0	0.03333	0.07104	0.07143	0
0	0	0	0.00546	0.10714	0.05

Table S3: The SNV of autophagy-related genes in pan-cancer

	ACC	BLCA	BRCA	CESC	CHOL	COAD	DLBC	ESCA
ULK1	0.01087	0.03155	0.00304	0.01384	0	0.06015	0	0.01087
ULK2	0	0.01942	0.00913	0.0173	0	0.05764	0	0.01087
ATG2A	0.01087	0.04126	0.01318	0.04152	0.01961	0.07519	0.02703	0.03261
ATG2B	0.02174	0.03883	0.01014	0.02768	0	0.07018	0.02703	0.02174
ATG3	0.01087	0.00728	0.00406	0.01384	0	0.00752	0	0.01087
ATG4A	0.01087	0.00728	0.00609	0.00692	0	0.01754	0	0
ATG4C	0.01087	0.01942	0.00203	0.01384	0	0.01253	0.05405	0
ATG4D	0.01087	0.02184	0.0071	0.01038	0	0.03008	0	0
ATG5	0	0.00971	0.00406	0.0173	0	0.02005	0	0.01087
BECN1	0.01087	0.01214	0.00101	0.00692	0	0.01003	0	0
ATG7	0	0.01699	0.0071	0.02076	0.01961	0.02005	0	0.02717
GABARAPL	0.02174	0	0.00101	0.00346	0	0.01003	0	0
GABARAPL	0	0	0.00101	0	0	0.00752	0	0.00543
MAP1LC3/	0	0.01456	0	0.01038	0	0.01253	0	0
MAP1LC3(	0	0.00728	0.00203	0	0	0.01253	0	0.00543
MAP1LC3I	0	0.00243	0	0.00346	0	0.00752	0	0.00543
GABARAP	0	0.01456	0.00304	0.00692	0	0	0	0
ATG9A	0	0.01456	0.00507	0.0173	0	0.03008	0	0.0163
ATG9B	0	0.00485	0.00203	0.02076	0	0.03759	0.02703	0.00543
ATG10	0	0.00243	0	0.00692	0	0.01754	0	0
ATG12	0	0.00485	0.00203	0.00692	0	0.01253	0	0.00543
ATG13	0	0.01214	0.00304	0.02076	0	0.01253	0	0
ATG14	0	0.01699	0.00203	0.01038	0.01961	0.01003	0	0.00543
ATG16L1	0	0.01214	0.00811	0.02422	0	0.01504	0	0
ATG16L2	0	0.00728	0.00609	0.0173	0	0.01253	0	0.00543
RB1CC1	0	0.04612	0.0142	0.04498	0.05882	0.03759	0.02703	0.02717
WIPI1	0	0.01214	0.00507	0.00692	0	0.01504	0	0.00543
WIPI2	0	0.00728	0.00507	0.02422	0.03922	0.02256	0	0
ATG101	0	0.00485	0.00304	0.00346	0	0.01003	0	0

GBM	HNSC	KICH	KIRC	KIRP	LAML	LGG	LIHC	LUAD
0.00769	0.01581	0	0.00595	0.01068	0	0.00198	0.00824	0.02139
0.01538	0.00395	0	0.00298	0.00712	0.00746	0.00395	0.01374	0.01783
0.00513	0.01581	0.0303	0.0119	0.01068	0	0.00791	0.00549	0.02852
0.01026	0.03162	0.01515	0.00595	0.01779	0	0.00593	0.02473	0.04278
0	0.00198	0	0.00298	0	0	0	0.00549	0.01248
0	0.00395	0	0.00298	0	0	0.00198	0.00824	0.0107
0.00256	0.00198	0	0.00595	0.00712	0.00746	0	0.00549	0.00178
0.00769	0.00791	0	0.00298	0.00712	0	0.00395	0.00275	0.01248
0.00256	0	0	0	0	0	0.00593	0.00275	0.00535
0.00256	0.00395	0	0.00298	0	0	0.00198	0.00275	0.0107
0	0	0.01515	0.0119	0.01068	0	0.00198	0	0.01248
0.00513	0	0	0	0	0	0	0	0.00357
0.00256	0.00395	0	0	0	0	0	0	0
0	0.00198	0	0.00595	0	0	0	0.00275	0
0	0.00395	0	0	0	0	0	0	0.01783
0.00256	0	0	0.00298	0	0	0	0	0.00178
0	0	0	0	0	0	0	0.00549	0
0.00256	0.00395	0	0	0.01068	0	0.00198	0.00275	0.01248
0	0.00198	0	0	0.00356	0	0.00395	0.00824	0.0107
0	0.00198	0	0	0.00356	0.00746	0	0.00275	0.00357
0.00513	0.00198	0	0.00298	0.00356	0	0	0.00275	0
0.00513	0.00791	0	0.00595	0.00712	0	0.00198	0.00275	0.00891
0.00256	0.00593	0	0	0.00356	0.00746	0	0.01099	0.00357
0.00256	0.00988	0	0.00298	0.00356	0.01493	0.00198	0.01099	0.01426
0.00513	0.00395	0	0.00298	0	0	0.00395	0.00549	0.00535
0.02308	0.01779	0.01515	0.0119	0.01423	0	0.00593	0.01648	0.03387
0.00769	0.00593	0	0.00298	0.00356	0	0.00198	0	0.00713
0.00769	0.00395	0.01515	0.00595	0.00712	0	0.00791	0	0.00891
0.00513	0.00198	0	0	0	0	0.00198	0	0.00357

LUSC	MESO	OV	PAAD	PCPG	PRAD	READ	SARC	SKCM
0.0224	0	0	0.01266	0	0.00413	0.02206	0.01688	0.05996
0.01222	0	0.00229	0	0	0.00207	0.02941	0.00844	0.0364
0.03462	0	0.00917	0.01899	0	0.0062	0.04412	0.00422	0.08779
0.03666	0	0.00917	0.01266	0.00562	0.00413	0.03676	0.0211	0.06638
0.01426	0	0.00688	0.00633	0	0.00207	0.01471	0.00422	0.00857
0.01018	0	0.00229	0	0	0	0.01471	0	0.02141
0.02037	0	0.00229	0	0	0	0.02206	0	0.01499
0.01222	0	0.00459	0	0	0	0.01471	0	0.01499
0.00407	0	0	0	0	0.00413	0.00735	0.00422	0.00857
0.01222	0	0.00459	0.00633	0	0.00207	0.01471	0.00422	0.01927
0.02851	0	0.00229	0.00633	0	0.00207	0.00735	0.00844	0.02998
0.00204	0	0	0.00633	0	0	0	0	0
0.00407	0	0	0.00633	0	0	0.01471	0	0
0	0	0	0.01266	0	0	0	0	0.00642
0.01629	0	0.00688	0.00633	0	0.00207	0	0	0.01071
0	0	0	0	0	0	0	0	0.00214
0.00204	0	0	0	0	0	0	0	0.00428
0.01222	0	0.00459	0.01266	0	0.00413	0.02941	0	0.03212
0.01629	0	0.00459	0.00633	0	0	0.02206	0	0.0364
0.00611	0	0	0.00633	0	0.00207	0.01471	0.00422	0.00428
0.00815	0	0.00229	0	0	0.00207	0	0	0.01285
0.01222	0	0	0	0	0.0062	0.01471	0.01266	0.01499
0.00815	0.0125	0.00459	0.01266	0	0.0062	0.01471	0.00422	0.01285
0.01426	0	0.00459	0	0	0.00207	0.01471	0.00422	0.03212
0.00611	0	0.00229	0.01266	0	0	0.00735	0	0.01285
0.03055	0	0.02294	0.01266	0	0	0.07353	0.00844	0.03854
0.00407	0	0	0.01266	0	0.00207	0.02206	0	0.00857
0.00611	0	0.00688	0.01266	0	0	0.01471	0.00422	0.02355
0	0	0.00459	0.00633	0	0	0.00735	0.00422	0.00642

STAD	TGCT	THCA	THYM	UCEC	UCS	UVM
0.05774	0.0069	0	0	0.07372	0.01754	0.0125
0.02771	0	0	0	0.0775	0	0
0.06236	0.0069	0.00205	0	0.09641	0.01754	0
0.03695	0.01379	0.00411	0	0.10397	0.01754	0
0.00231	0	0	0	0.0397	0	0
0.00924	0	0	0	0.05293	0.01754	0
0.01155	0	0	0	0.05671	0.01754	0
0.01155	0	0	0	0.02647	0	0
0.00924	0	0	0	0.0397	0	0
0.0254	0	0	0	0.04726	0	0
0.01848	0	0	0.01639	0.09452	0	0
0.01386	0	0	0	0.04159	0	0
0.01155	0	0	0	0.0189	0	0
0.00924	0	0	0	0.02079	0	0
0.01155	0	0	0	0.03214	0	0
0.00231	0	0	0	0.01701	0.01754	0
0.00924	0	0	0.0082	0.0189	0	0
0.0254	0	0	0.0082	0.06616	0	0.0125
0.02079	0	0	0	0.05671	0.01754	0
0.00924	0	0	0	0.02836	0	0
0.00693	0	0.00205	0	0.01323	0	0
0.01848	0	0	0.0082	0.03403	0.01754	0
0.01617	0	0.00205	0.0082	0.0586	0	0
0.01386	0	0	0	0.09074	0	0
0.01155	0	0.00205	0	0.05293	0.01754	0
0.04388	0	0.00205	0.01639	0.13989	0.01754	0
0.00462	0	0	0	0.04348	0	0
0.02771	0	0	0.01639	0.09641	0	0
0.00924	0	0	0	0.01512	0	0

Table S4: logFC of autophagy-related genes across cancer types

	BLCA	CHOL	COAD	ESCA	KICH	KIRC	KIRP	LIHC
ATG16L2	0.43503	1.17422	0.38052	-0.0223	-0.3949	2.37673	1.31042	1.02505
ATG2B	-0.1915	0.6828	-0.2834	-0.1821	-0.0698	-0.4141	-0.3561	0.16944
ULK2	-0.2805	2.24619	-0.1658	-0.2041	-0.9477	-0.0526	0.09279	0.48366
ATG10	0.17568	1.19779	0.23162	0.3215	-0.4897	0.0813	0.11742	1.02262
ATG14	0.11988	1.64076	0.00289	0.1708	0.07351	0.07214	0.33948	0.98383
WIPI1	0.42749	2.01595	-0.355	0.07367	-0.118	0.01925	1.10757	0.94118
WIPI2	0.69715	1.03605	0.52285	0.33305	0.98984	-0.1024	0.71026	0.82056
ATG7	0.57368	1.29847	0.09433	0.0853	-0.1444	0.06517	0.38026	0.8548
ATG4C	0.1388	0.71449	-0.1313	0.28722	-0.9883	-0.0599	-0.4739	0.51438
BECN1	-0.0163	1.68917	-0.1527	0.00035	-0.8289	-0.4401	-0.0622	0.71303
GABARAP	-0.1941	1.05712	-1.0552	0.04942	-1.005	0.02407	0.18423	0.2291
ATG101	0.10661	1.25893	0.56525	0.48797	0.38051	-0.0791	-0.055	0.24112
MAP1LC3A	-0.2894	0.0875	0.42816	0.01043	0.65192	-0.342	-0.3106	0.23226
ATG4A	0.14489	0.32008	-0.7232	-0.1433	0.46141	-0.1468	-0.4923	0.50356
GABARAPL	-0.9614	-0.0606	-0.7957	-0.9605	0.61506	-1.5556	-0.9512	-0.4804
ATG13	0.1701	1.55639	-0.1518	0.17095	-0.1182	-0.1355	-0.0749	0.88981
MAP1LC3I	-0.2583	1.34766	-0.1163	0.39412	0.05226	-0.07	0.68886	0.34582
ATG5	0.24543	0.40203	-0.0696	0.32788	-0.6952	-0.4812	-0.1909	0.25201
ATG16L1	-0.0966	1.39792	0.53448	0.26266	-0.7837	0.96662	0.57155	0.55456
ATG12	0.22599	1.16513	0.32426	0.22352	-0.1363	1.02031	0.46394	0.8206
ATG9A	0.24289	1.0149	-0.1024	0.15363	-0.5119	-0.0131	0.25873	0.72423
GABARAPL	0.08363	1.29535	-0.2174	0.18913	-0.3687	-0.468	-0.1258	0.55837
ULK1	0.45757	1.50942	0.26722	0.03116	1.09376	0.55442	0.22125	0.91979
ATG2A	0.25259	0.11182	-0.151	0.26666	0.48502	-0.2679	-0.2426	-0.1544
RB1CC1	0.08147	0.99536	0.4545	0.36516	0.3973	-0.0729	-0.1717	1.0142
ATG3	0.35838	1.13504	0.30837	0.49173	0.00942	-0.0634	######	0.73036
MAP1LC3C	-1.0085	1.30989	-1.7434	-2.4659	-2.3041	0.66082	2.59734	1.07551
ATG9B	1.70088	3.71201	3.7342	-0.2787	0.87563	2.95225	0.9768	1.05237
ATG4D	0.36053	2.24957	-0.6601	0.50394	1.09218	-0.2876	0.0526	0.92044

LUAD	LUSC	PAAD	PCPG	PRAD	READ	SARC	SKCM	STAD
0.12651	-0.4621	-0.5104	-0.521	0.42728	0.26346	1.06582	-0.8242	0.3427
-0.0811	-0.2884	0.08053	0.5114	0.14032	-0.4366	-0.1302	0.18282	0.28348
-0.7693	-0.6417	-0.3631	0.06501	-0.1043	-1.2682	0.17324	0.03314	-0.3325
0.2673	0.27418	-0.1773	0.7784	0.07895	-0.2842	-0.1238	-0.0147	0.32985
0.46676	0.35138	0.19231	-0.143	-0.141	-0.0622	0.06241	0.10739	0.22753
0.46748	0.82949	-0.3663	-0.2488	0.07764	-0.3892	0.28824	0.66929	0.31858
0.51567	0.54055	-0.0431	0.39712	0.23692	0.51656	-0.07	0.18772	0.367
-0.1524	-0.5356	-0.2754	-0.389	0.04769	0.08738	0.96762	-0.2425	0.40443
0.14326	-0.182	0.0147	-0.3701	0.04376	-0.5408	0.06117	-0.563	0.37866
0.36644	0.17868	0.10243	-0.4609	-0.0054	-0.3576	-0.5454	0.10558	0.02013
-0.2454	-0.4752	-0.6968	-0.3872	-0.0403	-1.1975	-0.0016	-0.1696	-0.2656
0.37611	0.61973	-0.1514	-0.4015	0.22912	0.63816	0.75802	0.34093	0.22396
-0.2044	-0.2145	-0.2064	1.40328	0.20282	0.54889	-0.1114	0.24036	-0.1538
0.01201	-0.0546	0.15284	0.63513	0.2095	-0.7607	-0.7424	0.08329	-0.1925
-0.89	-0.5864	-0.6003	0.38199	-0.326	-1.5147	-2.4751	0.10249	-0.9238
0.25386	0.44096	0.0469	0.49318	0.1505	-0.0614	-0.1156	0.16906	0.19155
-0.2112	-0.4421	-0.0911	0.23878	-0.505	-0.3797	0.27356	0.21611	0.1837
0.11367	0.58276	0.10861	-0.7451	-0.4887	-0.134	0.22677	-0.0258	0.4972
0.77737	0.45319	0.40732	0.20172	0.05022	0.41718	0.16742	0.00445	0.50516
0.30547	0.11601	0.23907	0.55627	-0.2415	0.07554	1.51324	-0.1051	0.20035
0.34994	0.43405	-0.1241	0.09319	0.12362	0.28219	0.39402	0.66314	0.23692
-0.251	-0.3009	0.0101	0.68515	0.01116	-0.4063	-0.6273	-0.5067	-0.0647
0.73663	1.10607	-0.3307	0.44673	0.25035	0.11873	0.51152	0.64732	-0.0717
0.39056	0.10762	0.16544	-0.0024	0.11698	0.31162	-0.2192	0.54513	0.54932
0.16468	0.47458	0.18763	-0.077	0.02403	0.2517	0.04248	0.55525	0.48751
0.07741	0.49651	0.05264	-0.4631	-0.0381	0.24368	0.25468	-0.8325	0.33376
-1.1943	-3.5445	-0.4351	-1.286	-0.0911	-1.2689	6.77337	-2.0323	-0.6515
1.99042	1.96692	1.47274	4.32407	1.5751	3.3271	2.31624	0.04338	-1.2187
0.45076	1.38802	-0.0294	-0.2746	0.34949	-0.0251	-1.0919	0.4674	-0.3091

THCA	THYM	UCEC
0.06196	-0.2437	0.26091
-0.5242	-0.653	-0.6798
-0.136	-0.4563	-0.8607
0.06764	-0.1009	0.46865
-0.2297	-0.2587	-0.5553
-0.0425	-0.8747	1.1493
0.03569	0.01279	0.07688
0.12049	0.13538	0.25044
-0.5045	0.0035	-0.6073
0.14301	-0.4494	-0.0802
0.08952	0.0954	-0.4521
0.10485	0.01198	0.6357
-0.816	-0.0663	0.45202
-0.1821	0.13041	0.46272
-0.4855	-0.5387	-1.5966
-0.178	0.13901	-0.0154
0.09274	-0.2201	-0.4906
-0.1769	-0.1265	-0.0703
0.00303	-0.4128	0.09987
-0.072	-0.1167	-0.1409
0.2862	-0.5357	0.38717
-0.0205	0.25092	-0.715
-0.0227	-0.3023	-0.2345
0.19686	-0.4852	-0.0091
-0.273	-0.4577	-0.4116
-0.1457	0.12524	0.50384
-1.2762	-2.0079	0.4246
1.00398	0.34145	0.54597
-0.0045	-0.1955	1.32184

Table S5: —logP value of changes in mRNA expression

	BLCA	CHOL	COAD	ESCA	KICH	KIRC	KIRP	LIHC
ATG16L2	0.34704	6.83E-05	0.0182	0.54209	0.00145	5.17E-32	2.36E-08	2.00E-13
ATG2B	0.02233	0.09054	7.96E-07	0.1668	0.37729	1.22E-13	2.39E-05	0.46058
ULK2	0.025	2.85E-06	0.08668	0.66737	1.14E-09	0.09213	0.7595	0.74238
ATG10	0.39235	1.47E-07	0.00583	0.11818	1.01E-05	0.1388	0.23036	1.11E-22
ATG14	0.44331	4.19E-08	0.49175	0.48209	0.68078	0.68092	0.00021	1.06E-19
WIPI1	0.02664	2.01E-06	1.45E-06	0.98671	0.11711	0.96003	8.71E-13	1.77E-07
WIPI2	3.48E-08	2.51E-07	6.90E-14	0.00478	1.71E-09	1.30E-08	2.17E-13	1.76E-23
ATG7	0.00022	2.09E-08	0.26987	0.49886	0.24221	0.76393	1.82E-06	1.89E-22
ATG4C	0.3578	0.01091	0.07914	0.15681	1.21E-09	0.12672	8.96E-12	3.97E-09
BECN1	0.77845	1.40E-06	0.00016	0.92833	2.85E-09	9.44E-20	0.63926	4.76E-11
GABARAP	0.03815	1.47E-07	4.16E-19	0.48209	3.05E-12	0.4151	0.01172	0.00548
ATG101	0.21969	1.47E-07	6.97E-11	0.00206	0.00022	0.01639	0.23428	0.00863
MAP1LC3/	0.01373	0.68659	0.03928	0.339	0.00123	5.91E-12	4.87E-05	0.34689
ATG4A	0.39235	0.09723	5.95E-16	0.34578	1.90E-05	2.02E-07	4.02E-12	5.97E-09
GABARAPL	2.81E-07	0.32228	1.36E-15	0.01987	0.00011	3.00E-37	1.11E-14	8.06E-09
ATG13	0.26364	2.09E-08	0.00017	0.17726	0.10273	2.48E-05	0.02182	1.10E-25
MAP1LC3I	0.0019	1.27E-05	0.01276	0.00348	0.32482	0.07504	2.75E-10	0.01273
ATG5	0.13138	0.0575	0.15039	0.01061	5.31E-08	4.59E-26	0.00341	0.00165
ATG16L1	0.14336	4.19E-08	8.89E-17	0.2644	5.69E-10	2.85E-22	1.27E-10	3.39E-12
ATG12	0.26933	2.09E-08	0.00015	0.1882	0.03794	4.17E-36	3.74E-07	1.68E-22
ATG9A	0.05434	1.47E-07	0.01243	0.18376	2.55E-07	0.34938	0.00021	3.76E-17
GABARAPL	0.71695	2.09E-08	0.00012	0.31276	0.00095	2.48E-23	0.10288	5.07E-13
ULK1	0.00446	1.40E-06	0.00787	0.40304	3.15E-10	1.17E-09	0.07066	1.58E-15
ATG2A	0.0321	0.40886	0.01334	0.2644	3.97E-05	1.49E-09	3.76E-06	0.03904
RB1CC1	0.85023	0.00153	0.01005	0.12956	0.02108	0.00216	0.00909	4.73E-14
ATG3	0.0019	2.09E-08	1.33E-06	0.00085	0.93736	0.03024	0.93201	5.80E-19
MAP1LC3(	4.41E-05	0.42023	1.77E-11	0.0097	2.72E-10	0.00095	1.42E-05	0.8444
ATG9B	0.00081	8.37E-08	1.78E-23	0.03496	0.59501	2.52E-29	0.8827	5.60E-06
ATG4D	0.10684	2.09E-08	1.48E-12	0.01952	1.75E-11	7.97E-12	0.9384	2.56E-12

LUAD	LUSC	PAAD	PCPG	PRAD	READ	SARC	SKCM	STAD
0.84058	0.00027	0.15117	0.08356	0.00192	0.87478	0.36735	0.16318	0.03046
0.00816	4.47E-06	0.84329	0.08593	0.14562	0.08822	0.61224	0.76909	0.02226
1.63E-19	4.25E-14	0.21265	0.99475	0.07941	0.01953	0.89796	0.8891	0.18744
0.00441	0.02188	0.4756	0.02321	0.27396	0.43071	0.65306	0.84292	0.00263
2.95E-08	1.64E-05	0.50808	0.34677	0.00666	0.69889	0.97959	0.97658	0.04328
1.49E-08	1.87E-11	0.16641	0.2337	0.6501	0.1325	0.77551	0.90652	0.03925
2.77E-17	2.24E-18	0.67364	0.03249	2.89E-07	0.04644	0.44898	0.76909	4.66E-06
0.00014	9.57E-17	0.10799	0.10697	0.68786	0.8974	0.20408	0.37848	5.84E-06
0.22591	0.00223	0.96572	0.04478	0.55684	0.1325	0.97959	0.20682	8.82E-05
1.37E-11	0.0024	0.51916	0.15336	0.85705	0.07804	0.20408	0.91816	0.7728
3.17E-06	9.56E-15	0.02718	0.04478	0.45511	0.03052	0.89796	0.44969	0.00318
4.62E-06	1.94E-13	0.46503	0.10986	0.00139	0.05672	0.4898	0.61773	0.10146
6.12E-05	2.30E-06	0.49713	0.00801	0.44044	0.48267	0.73469	0.97073	0.07787
0.31627	4.33E-05	0.49713	0.02751	0.00049	0.04049	0.12245	0.92399	0.01485
1.75E-23	2.47E-15	0.0434	0.14961	1.08E-07	0.01953	0.04082	0.99414	2.19E-06
4.91E-07	1.84E-11	0.95202	0.14961	5.38E-05	0.67779	0.65306	0.79162	0.02579
5.20E-07	1.67E-14	0.50808	0.34007	3.17E-20	0.1735	0.69388	0.7747	0.05685
0.40487	1.66E-12	0.62419	0.00387	3.45E-08	0.55694	0.65306	0.83145	2.63E-06
1.57E-27	2.84E-11	0.18278	0.47327	0.99248	0.06458	1	0.65968	2.08E-08
2.55E-05	0.13683	0.16121	0.01883	2.54E-05	0.78546	0.04082	0.79728	0.02556
1.50E-09	1.52E-09	0.38533	0.95278	0.00213	0.38216	0.32653	0.16541	0.02237
4.51E-07	3.67E-09	0.93834	0.01312	0.32159	0.10546	0.08163	0.18405	0.73525
8.67E-12	2.04E-20	0.34882	0.30781	2.92E-05	0.98857	0.93878	0.21758	0.52786
5.40E-07	0.51747	0.51916	0.84868	0.04117	0.36675	0.4898	0.44093	1.01E-05
0.97308	8.74E-06	0.68623	0.69787	0.52133	0.85225	0.81633	0.52793	7.50E-05
0.38734	3.74E-11	0.88383	0.03701	0.48525	0.35173	0.65306	0.10325	0.00046
3.40E-16	1.77E-29	0.81648	0.02393	0.15413	0.05493	0.12245	0.12325	0.00199
6.82E-11	5.24E-12	0.37599	0.00457	7.03E-16	0.01953	0.2449	0.49027	6.40E-07
1.26E-07	1.00E-24	0.77669	0.19492	7.26E-10	0.55694	0.12245	0.39051	0.22814

THCA	THYM	UCEC
0.96666	0.36976	0.46249
7.49E-18	0.07072	2.70E-08
0.00071	0.35564	6.22E-11
0.74672	0.84085	8.23E-05
2.18E-05	0.25516	6.52E-07
0.1226	0.05558	5.69E-06
0.51422	0.94663	0.40745
6.99E-05	0.39902	0.02456
4.08E-19	0.98932	6.46E-06
2.22E-06	0.11728	0.46016
0.00934	0.42963	0.10575
0.01037	0.96796	0.00054
2.03E-17	0.98932	0.0472
5.18E-07	0.86184	0.00029
7.79E-17	0.36976	4.38E-12
8.98E-08	0.5831	0.8492
0.00344	0.24415	6.64E-06
2.09E-08	0.6394	0.23573
0.88753	0.25516	0.69878
0.0165	0.49477	0.08494
4.54E-16	0.03329	0.0002
0.74927	0.17635	8.56E-11
0.08628	0.10527	0.02337
2.05E-06	0.03799	0.94439
8.29E-09	0.15203	0.00028
1.61E-07	0.5831	2.75E-06
9.20E-19	0.0379	0.00496
7.77E-08	0.98932	0.00652
0.35408	0.67814	2.02E-11

Table S6: Hazard ratio of autophagy-related genes across cancer types

	ACC	BLCA	CHOL	COAD	DLBC	ESCA	KICH	KIRC
ATG5	0.96812	0.98879	1.0892	0.968	0.86831	1.11306	1.31018	1.0074
MAP1LC3/	1.0567	0.99823	0.97111	0.99328	0.98693	1.01811	0.94279	1.00508
ATG4A	0.90363	0.96989	1.13156	0.9763	1.1085	1.07091	1.1029	0.85056
ATG2B	0.95981	1.07952	1.08228	1.05427	0.82745	0.90119	1.02411	0.84239
ATG101	1.07402	1.00325	1.31056	1.00729	0.84218	1.06771	0.91878	1.00022
ATG16L1	1.38467	0.96844	1.04011	0.99312	1.00282	0.92964	1.46159	1.00811
WIPI1	0.99055	1.05174	1.02003	0.96088	0.8654	1.02253	1.31677	1.00557
MAP1LC3(	1.11297	1.29089	0.16761	15.097	0.92147	0.95961	135.507	1.08602
BECN1	1.02418	1.00524	0.98366	0.97722	0.92251	0.93896	1.13231	0.91236
GABARAPI	0.96397	1.00508	0.98639	1.01014	1.02536	1.06674	0.92855	0.96479
ULK2	1.30584	1.09883	0.8765	1.0487	0.60137	0.98596	1.32327	1.03252
ATG13	1.07577	1.00167	1.07446	1.08615	0.66447	1.00505	0.97176	1.06791
ATG4C	0.83461	1.03219	0.9785	0.98598	1.0675	1.08766	1.4742	0.80449
ATG10	0.93819	1.04396	0.58718	0.8833	0.72854	1.03145	7.03951	0.72425
ULK1	0.96675	0.9911	1.07367	1.17061	0.96871	1.00113	0.93565	1.05216
ATG2A	0.99721	1.00694	1.00988	1.03631	0.80037	0.97672	1.15256	0.95983
ATG9B	0.97637	1.0113	1.04988	1.03621	0.04051	0.96434	1.01832	1.0332
ATG9A	1.08585	1.06493	1.16072	1.0186	0.74437	0.99233	0.92747	1.04606
GABARAP	0.99043	1.0073	1.08388	1.02521	0.99884	1.04341	1.14279	0.98046
ATG14	1.103	0.97146	0.85585	1.03189	0.88306	1.03248	1.22429	0.93738
WIPI2	1.12625	1.01644	0.96925	1.09303	0.77959	0.9775	0.92138	1.01941
ATG16L2	1.33145	0.98112	0.65121	1.00811	1.25937	0.96137	1.87779	1.10464
ATG7	0.8808	1.02392	1.12423	0.79727	0.40744	1.06676	4.68543	0.93953
ATG3	1.16902	1.00693	0.97973	0.94372	0.72747	1.06897	1.33443	0.92585
GABARAPI	1.00164	1.00358	1.01138	1.07858	0.76221	1.02189	0.98878	0.99689
ATG4D	1.08791	0.98195	1.02824	0.94797	0.91764	1.00722	1.05891	1.00213
ATG12	0.98744	0.89086	1.09577	1.08783	0.81497	1.12945	2.67886	1.09405
RB1CC1	0.89099	0.98698	1.10093	0.98982	1.07838	1.03448	1.2832	0.96879
MAP1LC3I	0.98887	0.99895	0.98006	1.01512	1.09186	1.05495	0.91643	0.9617

KIRP	LAML	LIHC	LUAD	LUSC	MESO	OV	PAAD	PCPG
1.25814	0.98104	1.15715	1.01164	1.03056	1.05792	0.99932	0.94368	0.79902
0.94328	1.05514	0.99009	0.99481	1.00977	1.00296	1.004	0.96562	0.90027
0.84732	1.14781	1.00703	0.90679	0.96801	1.12166	0.94518	0.90288	1.38002
1.04645	0.90816	0.97316	0.91232	0.9555	0.76118	0.88468	0.92895	1.65167
1.06888	1.20024	1.11865	1.01047	0.99484	1.10248	0.99469	0.94731	1.13377
1.108	0.93458	1.23895	1.02958	0.9834	1.03217	1.04016	1.01429	1.05055
0.96133	0.96396	1.01864	0.96351	0.99394	1.09447	1.0086	0.97165	1.04238
1.00765	0.05279	1.04309	0.99143	1.19639	0.89706	0.94582	0.72202	3.33E-08
1.03037	1.10847	1.05174	0.98763	0.97629	0.94213	1.01236	0.98428	1.06902
0.98467	1.0217	0.99798	0.99382	0.99979	0.94129	0.99873	0.98295	1.06639
0.61383	1.04496	1.02232	0.99804	0.97913	0.88536	0.91303	0.86711	0.84868
1.02593	0.96735	1.08452	1.02756	1.008	0.94299	1.0059	0.96302	1.03631
1.24215	0.98841	1.17055	1.00247	0.98023	0.97054	1.01817	0.93852	0.61362
0.86348	0.86203	1.46541	1.21528	1.06508	0.90034	0.92655	0.94862	0.85884
1.00939	0.98852	1.0256	1.01206	1.01731	0.99287	1.00722	1.00714	0.95361
1.17517	1.06876	1.05766	0.95238	1.04405	0.89877	1.01479	0.96923	1.10531
1.22827	1.128	1.19734	0.92775	1.01103	3.83309	0.93217	1.10693	1.07224
1.11093	1.0073	1.09363	1.05271	1.00603	1.13964	1.02209	0.91115	0.90068
0.97384	1.01074	1.01305	0.98901	1.00872	0.97774	0.99999	0.95741	0.96433
0.93353	0.93541	1.16164	1.00376	0.96351	0.9159	1.05478	1.02491	1.14645
0.93147	0.95923	1.03051	0.99785	0.99442	1.02822	1.00942	0.88193	0.60739
0.85296	0.99637	0.99164	0.91561	0.97815	0.88584	1.01137	0.82695	0.25579
0.86595	0.97376	1.60908	1.0019	0.98073	0.84192	1.05471	0.98385	1.92568
0.92368	1.02786	1.1866	0.97814	0.98634	0.98627	0.9926	1.0571	1.08149
0.99238	0.96061	0.98181	1.00803	0.9995	0.98104	1.01429	0.99552	0.94925
0.85377	1.0228	1.0129	0.99891	0.99117	0.93915	1.00754	0.93362	1.37075
1.4159	0.87568	1.20839	1.11163	1.06831	1.01596	0.96568	1.08836	0.94417
1.2651	0.97369	1.02317	1.02321	0.99956	0.96233	0.9741	1.04393	1.10611
0.97958	1.02381	1.02528	0.99672	1.02177	0.95978	0.98064	0.98551	1.01931

PRAD	READ	SARC	SKCM	STAD	TGCT	THCA	THYM	UCEC
0.91813	0.91703	1.07338	0.93952	0.99991	1.03185	0.93014	0.75757	1.00419
1.02473	1.03669	0.98271	1.00008	1.00082	0.9072	1.03308	1.04096	0.99941
0.92789	1.15447	0.99881	0.9096	0.99046	1.1219	1.16475	1.24862	1.08764
1.15489	1.19821	1.16067	0.94972	1.10241	0.9605	1.56539	1.24547	1.19651
1.01216	1.04073	0.87501	0.99244	0.99047	0.97625	0.96478	0.91344	0.99223
1.49404	1.40869	1.2106	0.95282	0.94134	1.0086	0.92779	0.74706	1.14454
1.10169	0.91478	1.01639	1.00473	1.02495	2.07356	1.02178	1.19572	1.00901
5.31815	5.14E-05	1.00165	1.02252	1.01506	0.86016	1.03543	0.77597	1.04699
1.1747	0.78016	1.16695	0.96424	1.04877	1.11782	1.01138	1.05696	0.99467
1.063	1.0158	1.02314	0.97949	1.05195	1.12179	0.94541	1.06702	1.01747
1.2115	1.12643	1.19508	1.0064	1.0353	0.73905	1.14879	1.06771	1.13465
0.83715	0.93175	1.00919	0.99024	0.97372	1.04473	1.02574	0.99876	1.0158
1.09344	0.62018	1.05451	0.97082	0.98276	1.10044	1.07278	0.62761	1.0478
0.33359	0.09594	1.98997	0.90098	1.20963	0.47619	0.68097	0.78945	1.21671
1.00665	1.24974	0.99472	1.08579	1.00318	0.96509	1.24826	0.76637	1.07267
1.18593	1.10052	0.95259	1.03279	0.97336	1.10893	1.00979	1.1284	0.98664
1.5244	1.28384	1.97966	1.23812	0.98525	0.95174	1.10697	1.253	1.04171
1.08386	1.07103	1.04733	1.04252	0.96426	1.16325	0.78515	0.64226	1.05615
0.96668	1.00821	0.96978	1.00578	1.01625	1.09755	0.94915	1.14645	1.01062
1.28486	1.04311	1.20456	0.97514	1.01335	1.03695	1.21585	1.15397	0.92473
0.97737	0.98551	1.05927	1.04506	1.03602	0.73054	0.96022	0.7594	1.05235
1.55668	1.10136	1.04441	0.82607	0.93062	1.1319	0.96948	1.12973	0.86062
1.08901	0.19729	0.72902	0.85796	1.05739	1.94565	0.92734	1.84663	1.07113
1.40531	0.72552	0.98289	0.92213	0.99312	0.98377	0.81456	1.00605	0.97203
0.94877	0.9289	0.99933	0.97264	1.04192	0.99974	1.06015	1.08592	0.98127
1.06911	1.05705	1.06982	1.05196	0.95856	0.83889	0.88217	0.90359	1.00919
1.19397	0.92094	1.03431	0.94318	1.02602	0.79696	1.67196	0.63908	1.00244
0.92511	1.0088	1.06328	0.97193	1.02093	1.00555	1.09539	0.9242	1.03525
1.12309	0.99135	0.99845	1.00387	1.03847	1.19825	1.00649	0.86261	1.01829

UVM
1.24971
1.01746
0.91613
1.50909
1.02855
1.35694
1.03279
0.75306
1.16178
0.98438
1.64727
0.88671
0.96327
1.44795
0.73207
0.86326
6.29447
0.93248
0.97494
1.1068
0.82268
1.37431
0.3921
0.96128
0.79544
1.05694
1.58536
1.08442
1.00911

Table S7: —logP value of hazard ratio in pan-cancer

	ACC	BLCA	CHOL	COAD	DLBC	ESCA	KICH	KIRC
ATG5	0.42767	0.53581	0.4641	0.31428	0.53568	0.00261	0.02325	0.84021
MAP1LC3/	0.01379	0.81138	0.28165	0.56485	0.74195	0.29845	0.05388	0.6064
ATG4A	0.07914	0.5242	0.40343	0.7248	0.6124	0.22121	0.23215	0.00186
ATG2B	0.79068	0.43331	0.65787	0.74147	0.62751	0.25355	0.94127	0.01387
ATG101	0.01478	0.79198	0.03505	0.80026	0.20791	0.43289	0.40367	0.99467
ATG16L1	0.00114	0.57522	0.84169	0.91108	0.98792	0.15227	0.22979	0.55042
WIPI1	0.87269	0.00812	0.61146	0.20035	0.41411	0.519	0.00205	0.84613
MAP1LC3(	0.14297	0.22155	0.39285	0.00389	0.82685	0.95117	0.01992	0.01266
BECN1	0.79291	0.83121	0.86086	0.57768	0.53932	0.31158	0.19982	0.02157
GABARAPL	0.00617	0.57937	0.75068	0.58622	0.54758	0.00058	0.01771	0.00421
ULK2	0.2091	0.01461	0.43583	0.79486	0.42814	0.88357	0.31486	0.71001
ATG13	0.06192	0.93583	0.20913	0.02306	0.06442	0.86385	0.72588	0.02422
ATG4C	0.35636	0.63173	0.89207	0.89165	0.78389	0.40591	0.1135	0.00013
ATG10	0.79308	0.73846	0.43602	0.59967	0.76199	0.91491	0.00018	0.04155
ULK1	0.10413	0.58252	0.17971	0.00474	0.9208	0.94219	0.23509	0.00065
ATG2A	0.9787	0.87076	0.95128	0.45573	0.30391	0.42728	0.34199	0.54128
ATG9B	0.82447	0.77385	0.90423	0.75126	0.16377	0.58573	0.97956	0.27453
ATG9A	0.00051	0.0087	0.08547	0.62967	0.44715	0.76384	0.63927	0.11204
GABARAP	0.57006	0.42568	0.05126	0.12056	0.96502	0.11877	0.015	0.05999
ATG14	0.28485	0.54343	0.26202	0.7108	0.72978	0.63847	0.23619	0.12855
WIPI2	0.01994	0.40495	0.74943	0.03781	0.11502	0.63737	0.14137	0.3911
ATG16L2	0.01803	0.35178	0.26585	0.93885	0.32668	0.69728	0.12776	0.00015
ATG7	0.50689	0.60959	0.74738	0.19037	0.02422	0.71599	0.00959	0.56526
ATG3	0.0101	0.81199	0.88139	0.22847	0.06443	0.12487	0.0104	0.10286
GABARAPL	0.80475	0.71621	0.50621	0.05665	0.12229	0.35672	0.08613	0.32864
ATG4D	0.00346	0.24487	0.53287	0.0808	0.67898	0.81712	0.05704	0.92758
ATG12	0.88034	0.04911	0.74314	0.1879	0.59105	0.23419	0.00239	0.06166
RB1CC1	0.10397	0.53773	0.26561	0.72575	0.67542	0.09924	0.00464	0.33768
MAP1LC3I	0.51084	0.94701	0.69868	0.47227	0.31691	0.00494	0.06102	0.02529

KIRP	LAML	LIHC	LUAD	LUSC	MESO	OV	PAAD	PCPG
0.00294	0.80619	0.0006	0.55791	0.0133	0.36509	0.96095	0.32373	0.47786
0.02022	0.07125	0.1208	0.66763	0.36572	0.86293	0.40031	0.04059	0.03038
0.18522	0.05953	0.84269	0.01228	0.1808	0.22047	0.28079	0.27487	0.01121
0.73263	0.04665	0.75431	0.23102	0.59083	0.07213	0.13761	0.58976	0.11849
0.28635	0.10992	0.00014	0.5845	0.79092	0.00328	0.79455	0.2441	0.44404
0.09448	0.45544	0.00209	0.40688	0.69168	0.73984	0.4155	0.7824	0.78434
0.08425	0.44667	0.38492	0.11464	0.53763	0.00626	0.80233	0.49134	0.70208
0.159	0.28666	0.76347	0.70532	0.06466	0.21516	0.20745	0.16346	0.28891
0.49965	0.2861	0.14507	0.62025	0.40125	0.25576	0.75935	0.77659	0.74985
0.3929	0.51935	0.90913	0.41482	0.97755	0.00228	0.87026	0.45532	0.00625
0.00073	0.4364	0.8654	0.97651	0.69743	0.0013	0.17019	0.40831	0.77175
0.5818	0.17551	0.0021	0.26848	0.5779	0.0651	0.3769	0.5013	0.63195
0.1604	0.76539	0.04687	0.96204	0.69987	0.79581	0.72953	0.49867	0.44581
0.64828	0.32797	0.00686	0.06439	0.57339	0.66784	0.60314	0.88243	0.85132
0.79177	0.49155	0.17457	0.48047	0.14864	0.84154	0.56118	0.86225	0.59861
0.03143	0.08145	0.09154	0.1166	0.17516	0.1189	0.51467	0.63992	0.59706
0.00012	0.7456	0.62257	0.1921	0.83248	0.01551	0.25742	0.01081	0.53124
0.01071	0.83879	0.0001	0.07025	0.75738	0.01313	0.14772	0.08285	0.5587
0.06945	0.52416	0.27166	0.27807	0.33967	0.20623	0.99897	0.04206	0.40441
0.4007	0.13877	0.04708	0.91536	0.31983	0.28754	0.27513	0.72199	0.65344
0.04449	0.48616	0.16985	0.92728	0.81238	0.52451	0.59915	0.04989	0.05672
0.05949	0.56119	0.93557	0.02002	0.6055	0.45646	0.85071	0.02107	0.16677
0.34868	0.41009	6.42E-05	0.97965	0.79664	0.00376	0.49543	0.91635	0.27426
0.34876	0.02729	8.08E-07	0.55637	0.4541	0.66192	0.75477	0.53774	0.72866
0.3497	0.14321	0.00849	0.05754	0.9334	0.06138	0.27969	0.81407	0.05874
0.00062	0.25404	0.63227	0.96223	0.37636	0.29477	0.44572	0.15499	0.04159
0.00107	0.03765	0.00629	0.02946	0.27036	0.67357	0.54371	0.39583	0.87911
0.00034	0.25815	0.31299	0.19125	0.95791	0.50596	0.2195	0.33372	0.64814
0.18467	0.27293	0.16502	0.7959	0.08405	0.10633	0.17761	0.52902	0.73026

PRAD	READ	SARC	SKCM	STAD	TGCT	THCA	THYM	UCEC
0.52972	0.32246	0.2167	0.02895	0.99226	0.69857	0.57919	0.146	0.88178
0.33399	0.04502	0.50636	0.99268	0.93509	0.68342	0.24786	0.00299	0.95638
0.72886	0.4668	0.99288	0.02308	0.84654	0.71678	0.22007	0.04879	0.23447
0.54353	0.66765	0.20673	0.40393	0.18113	0.92617	0.06618	0.36279	0.26392
0.88061	0.68169	0.09405	0.44113	0.82706	0.88827	0.69553	0.51847	0.75998
0.00021	0.0415	0.21949	0.05025	0.29286	0.94655	0.65414	0.28557	0.00883
0.17967	0.2691	0.65531	0.10505	0.34702	0.02363	0.88207	0.10896	0.73888
0.8514	0.16502	0.75443	0.49517	0.95314	0.87586	0.8888	0.82642	3.03E-05
0.1733	0.04688	0.22106	0.05136	0.26716	0.73845	0.92524	0.66702	0.91488
0.07554	0.68028	0.63118	0.01215	0.0067	0.35937	0.05661	0.11859	0.47283
0.46777	0.79914	0.03521	0.86817	0.71044	0.6195	0.67212	0.85594	0.44702
0.12592	0.55546	0.91499	0.46457	0.19262	0.58999	0.81567	0.98573	0.7476
0.76712	0.19068	0.70854	0.33747	0.83451	0.7126	0.61718	0.05009	0.6889
0.20733	0.02527	0.21118	0.41349	0.45592	0.50412	0.41831	0.56724	0.32917
0.95618	0.07035	0.88098	0.00065	0.89677	0.61894	0.10051	0.1555	0.04181
0.43096	0.39726	0.80818	0.04609	0.30349	0.19247	0.95371	0.70911	0.80573
0.16235	0.40848	0.28103	0.02677	0.87342	0.69134	0.01656	0.65962	0.70819
0.59746	0.28263	0.47512	0.0035	0.061	0.52471	0.14613	0.24061	0.02878
0.31851	0.86595	0.11095	0.34098	0.39831	0.35493	0.07634	0.01225	0.40471
0.23342	0.86291	0.09514	0.37691	0.84054	0.71933	0.10106	0.42499	0.43353
0.8612	0.89615	0.06315	0.00394	0.3607	0.26424	0.7892	0.07263	0.37527
0.08438	0.7145	0.87063	0.00488	0.45846	0.71821	0.85928	0.6088	0.15313
0.89489	0.00148	0.09228	0.03656	0.6202	0.03384	0.8851	0.06336	0.54369
0.00952	0.02561	0.85815	0.0007	0.83041	0.80877	0.16824	0.97029	0.5755
0.35644	0.56016	0.97941	0.01811	0.01465	0.98519	0.19568	0.0778	0.61687
0.56492	0.14518	0.15386	0.00051	0.09742	0.49546	0.38337	0.14107	0.76206
0.60569	0.73082	0.41644	0.15914	0.66483	0.8031	0.07298	0.19381	0.98325
0.43131	0.90509	0.14137	0.06015	0.36795	0.96031	0.44106	0.49262	0.0418
0.24997	0.88647	0.97254	0.54821	0.00831	0.32164	0.91267	0.08227	0.56241

UVM
0.15105
0.24384
0.7746
0.08396
0.80805
0.10169
0.0242
0.09902
0.17572
0.63138
0.01626
0.03288
0.5616
0.38915
0.15338
0.01486
0.14739
0.20962
0.14685
0.22048
0.17214
0.2355
0.07187
0.7999
0.00087
0.50029
0.04521
0.07942
0.79754

Table S8: Hazard ratio of autophagy-related genes in KIRC patients

gene	conMean	treatMean	logFC	pValue
ATG13	13.696761	12.46898	-0.135492	2.48E-05
MAP1LC3C	0.4311419	0.681629	0.6608249	0.0009549
BECN1	9.3209445	6.870167	-0.440131	9.44E-20
ULK2	2.9577808	2.851936	-0.052573	0.09213
ATG14	5.0827147	5.343313	0.0721354	0.6809227
ATG4D	8.2661836	6.772018	-0.287636	7.97E-12
ATG12	2.4663375	5.002604	1.0203091	4.17E-36
ATG2B	4.1113271	3.085548	-0.414078	1.22E-13
ATG101	6.3654539	6.025683	-0.079139	0.0163926
ATG7	2.6767653	2.800453	0.0651697	0.7639348
ATG16L1	4.0027445	7.822406	0.9666229	2.85E-22
MAP1LC3A	11.749331	9.269289	-0.342048	5.91E-12
ATG4A	6.9493875	6.276983	-0.146814	2.02E-07
GABARAP	29.42785	29.92298	0.024072	0.4151037
ATG4C	5.2379046	5.024825	-0.059917	0.1267168
MAP1LC3B	21.015738	20.02039	-0.07	0.0750389
ATG16L2	0.6701011	3.480231	2.3767323	5.17E-32
RB1CC1	8.384635	7.971657	-0.072868	0.0021637
GABARAPL2	37.606847	27.18879	-0.467983	2.48E-23
WIPI2	9.4101947	8.765541	-0.102382	1.30E-08
GABARAPL1	84.360109	28.69902	-1.555559	3.00E-37
ATG5	10.07714	7.219311	-0.481153	4.59E-26
WIPI1	7.4855775	7.586146	0.0192535	0.960033
ATG9A	8.4129496	8.336881	-0.013104	0.3493849
ATG10	1.6394603	1.734497	0.0812965	0.1388027
ATG3	7.3579853	7.041524	-0.063423	0.0302419
ATG2A	5.175264	4.298318	-0.26786	1.49E-09
ATG9B	0.1123896	0.869842	2.9522461	2.52E-29
ULK1	5.8306296	8.562715	0.5544167	1.17E-09

Table S9: Univariate Cox analysis

id	HR	HR.95L	HR.95H	pvalue
age	1.0297	1.01588	1.04372	2.19E-05
grade	2.28263	1.84053	2.83093	5.73E-14
stage	1.92576	1.67699	2.21144	1.60E-20
T	1.97255	1.66095	2.34262	9.65E-15
M	4.49933	3.25415	6.22096	9.22E-20
riskScore	2.16893	1.88633	2.49388	1.60E-27

Table S10: Multivariate Cox analysis

id	HR	HR.95L	HR.95H	pvalue
age	1.030998	1.015693	1.046534	6.32E-05
grade	1.355069	1.058149	1.735304	0.016046
stage	1.87452	1.259581	2.789679	0.001951
T	0.749129	0.523444	1.07212	0.114285
M	1.130371	0.59713	2.139799	0.706644
riskScore	1.697592	1.421278	2.027625	5.27E-09
